# Electrochemical sensing of macromolecules based on molecularly imprinted polymers: challenges, successful strategies, and opportunities

**DOI:** 10.1007/s00216-022-03981-0

**Published:** 2022-03-12

**Authors:** Elisabetta Mazzotta, Tiziano Di Giulio, Cosimino Malitesta

**Affiliations:** Laboratory of Analytical Chemistry, Department of Biological and Environmental Sciences and Technologies (Di.S.Te.B.A.), University of Salento, via Monteroni, 73100 Lecce, Italy

**Keywords:** Molecularly imprinted polymers, Macromolecules, Protein, Electrochemical sensors

## Abstract

Looking at the literature focused on molecularly imprinted polymers (MIPs) for protein, it soon becomes apparent that a remarkable increase in scientific interest and exploration of new applications has been recorded in the last several years, from 42 documents in 2011 to 128 just 10 years later, in 2021 (Scopus, December 2021). Such a rapid threefold increase in the number of works in this field is evidence that the imprinting of macromolecules no longer represents a distant dream of optimistic imprinters, as it was perceived until only a few years ago, but is rapidly becoming an ever more promising and reliable technology, due to the significant achievements in the field. The present critical review aims to summarize some of them, evidencing the aspects that have contributed to the success of the most widely used strategies in the field. At the same time, limitations and drawbacks of less frequently used approaches are critically discussed. Particular focus is given to the use of a MIP for protein in the assembly of electrochemical sensors. Sensor design indeed represents one of the most active application fields of imprinting technology, with electrochemical MIP sensors providing the broadest spectrum of protein analytes among the different sensor configurations.

**Graphical abstract:**

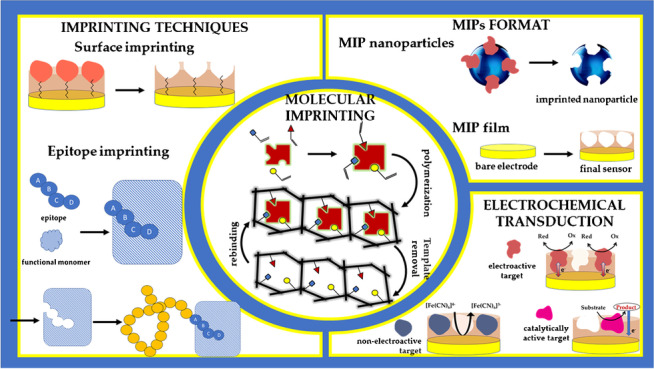

## Introduction

**Fig. 1:**
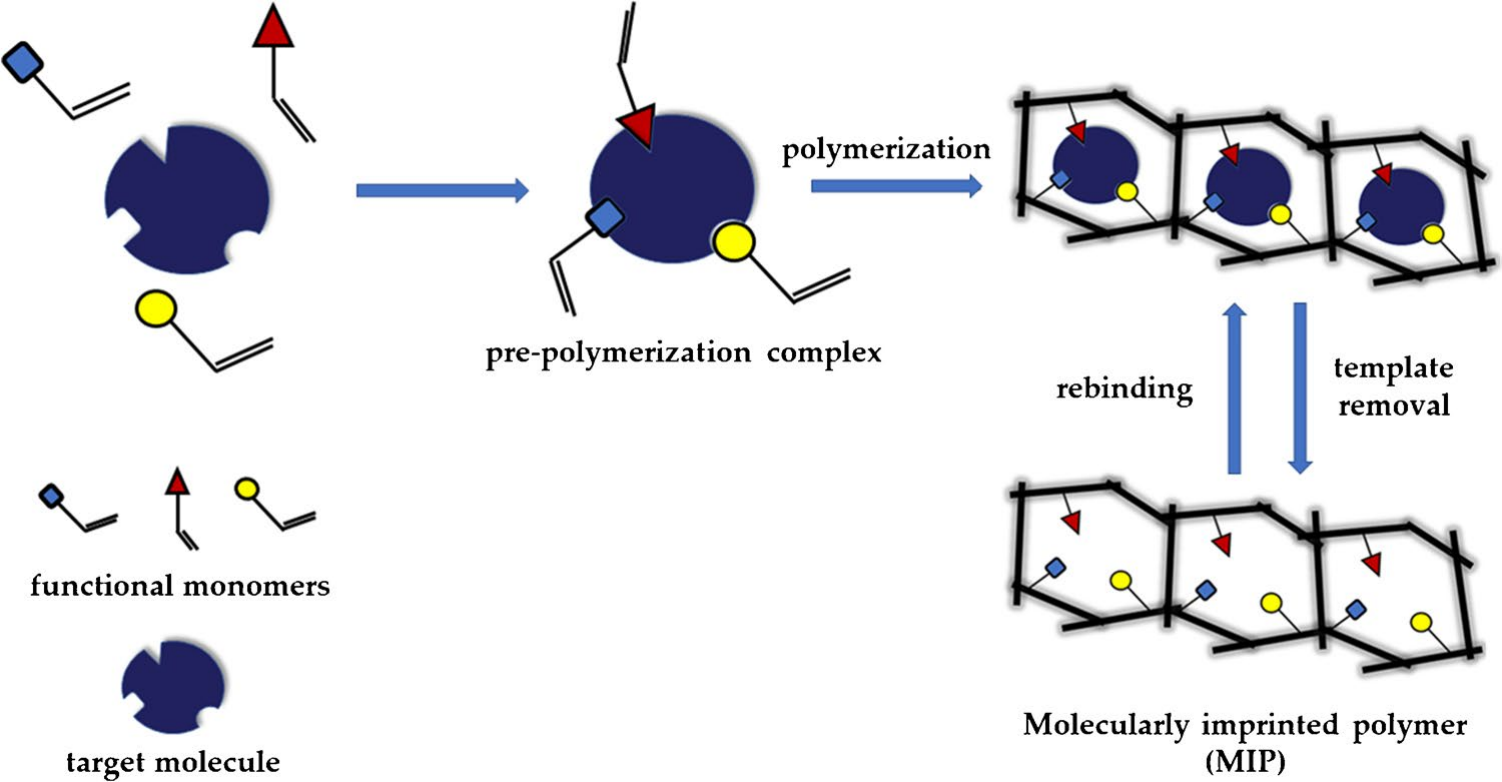
General scheme of imprinting process

In discussions about the great potential of molecularly imprinted polymers (MIPs), most scientists have certainly observed the great interest or even wonder raised in the audience, especially among those not specialized in the field. Indeed, someone first hearing about molecular imprinting technology is impressed by its ability to effectively create “artificial antibodies” that can be easily synthesized and engineered in the laboratory for highly selective matching of any target molecules. The enormous interest attracted by MIPs can be attributed to their ability to serve as an alternative to common bioreceptors, keeping their selectivity but possessing high stability, low cost, tailored fabrication for any target analyte, and easy synthetic schemes, in contrast to their natural counterparts. Such bioreceptor mimics are obtained by simply polymerizing the functional monomer(s) in the presence of the target analyte (template molecule), with subsequent removal of the template which thus leaves binding cavities within the polymeric network corresponding to the shape, size, and functionality of the template (Fig. 1).

Nonetheless, such high potential can find effective application in different fields—from sensing to separation science and from drug delivery to imaging—only when a correct imprinting scheme is designed. This can be achieved taking into account the functionalities of both monomer and template and selecting the optimal experimental conditions for MIP synthesis. Controlled polymer growth has to be enabled, leaving unaltered template moieties responsible for the interaction with the forming polymer and, subsequently, with MIP cavities. All these issues can become particularly critical when large macromolecules have to be imprinted. The imprinting of macromolecules indeed represented a challenge until about 10–15 years ago. From its beginnings [1–3], it was found that the classical bulk methodologies, which were effective for low-molecular-weight compounds, generally failed to address the peculiarities of macromolecular targets. As has been widely recognized [4], this was mainly due to the intrinsic complexity of macromolecules as proteins/peptides. Firstly, these molecules have a multitude of recognition sites on their surface, such as charged amino acids and hydrophobic and hydrophilic moieties, which can lead to cross-reactivity of MIPs with other similar targets. In addition, the interaction of macromolecules with the imprinted cavities may suffer from restricted diffusion, slow binding kinetics, and difficult access to binding sites. Also, the removal of macromolecules can be laborious due to their inclusion in the polymer matrix, while the large imprinted sites created by the macromolecular template can act as “nanopores,” possibly binding smaller molecules, leading to reduced selectivity [5, 6]. Finally, high macromolecular fragility can cause irreversible conformational changes during polymer growth, resulting in reduced ability to recognize their native form upon rebinding.

During recent years, considerable efforts have been made by researchers to address such issues, and excellent results have been achieved in macromolecule imprinting, especially in sensing applications, as reported in recent review papers [4, 7–11]. Such a significant heightening of scientific interest is certainly promoted by the growing need to develop selective detection schemes for macromolecules acting as “markers” in several analytical fields, including clinical diagnosis and food and environmental analysis [12, 13].

The present review aims to describe, albeit not exhaustively, some significant results presented in the field, with a special focus on MIP-based electrochemical detection of macromolecules. The choice to consider electrochemical transduction stems from its widely reported use in macromolecular detection, for which various successful strategies have been implemented, which will be critically presented and discussed herein.

In particular, three main topics will be covered, namely (1) a description of the mostly commonly used approaches in the electrochemical imprinting of macromolecules, (2) a survey of MIP formats exploited in the electrochemical detection of macromolecules, and (3) the strategies used for the generation of the electrochemical signal upon MIP–macromolecule interaction. Emphasis is also placed on the use of nanotechnology in this field, which represents a distinctive result which over recent decades has certainly promoted the wide application of imprinting technologies for macromolecular (electrochemical) detection.

## Approaches for imprinting of macromolecules in electrochemical sensor design

The strategy of using a solution containing functional monomer(s) and template, along with cross-linking agents (when required, to guarantee the desired rigidity of the resulting MIP), traditionally used for small-molecule imprinting has been similarly applied to macromolecule imprinting in the design of electrochemical sensors, using different strategies for integrating MIPs with electrode surfaces, as described in section 2.

Although interesting results have been achieved in the electrochemical detection of proteins using this approach [14], as discussed extensively below, it was revealed to suffer from limitations regarding the imprinting of larger and less flexible proteins. This is mainly due to the difficulties in controlling the orientation of the template and preserving its native conformational state during MIP formation, possibly leading to reduced MIP selectivity and sensitivity [15]. Moreover, the rebinding of large native protein, as well as its removal after polymerization, may be hindered. Thus, to address the need for establishing more controlled imprinting and to achieve easier removal/access of bulky macromolecules, alternative methods like surface imprinting and epitope imprinting have been introduced [10, 16, 17].

### Surface imprinting

Surface imprinting is, to date, among the most widely used approaches for imprinting of macromolecules and proteins for the development of electrochemical sensors. This methodology proposes to imprint the template only to the surface of the MIP membrane or to a very thin polymer layer whose thickness is comparable to the size of the protein template [4, 16]. This approach bypasses the need for the target to permeate the MIP matrix in order to reach the binding sites and trigger the molecular recognition event, and thus enables much faster binding kinetics, along with easier formation of imprinted cavities upon target removal. The potential of such a strategy has been demonstrated by its application not only to macromolecular imprinting [18, 19], but also to cells and microorganisms [20, 21].

In order to confine the templated sites exclusively to the polymer surface, several techniques have been exploited, such as soft lithography [6, 22, 23], micro-contact imprinting [24–26], and sacrificial template support methods [27, 28]. In this respect, in the field of electrochemical sensing, one alternative is the deposition of MIP layers directly on the electrode surface, which represents a suitable approach for achieving ultrathin polymers only partially embedding the target protein. As illustrated in detail in section 2.1, this can be obtained by drop-casting the pre-polymerization mixture onto the electrode followed by polymerization [29, 30]. Improved control of the thickness of the MIP layer can be obtained by surface-confined polymerization methods, where either the initiator [31, 32] or a polymerizable group [33] is attached to the surface. Also, MIP electropolymerization can be successfully used for this purpose, particularly with nonconductive polymeric layers self-limiting their deposition, resulting in films of few-nanometer thickness [34, 35]. In general, the possibility for fine-tuning the thickness of electropolymerized films by controlling experimental conditions (deposition time, circulated charge, etc.) is certainly beneficial for this purpose.

**Fig. 2:**
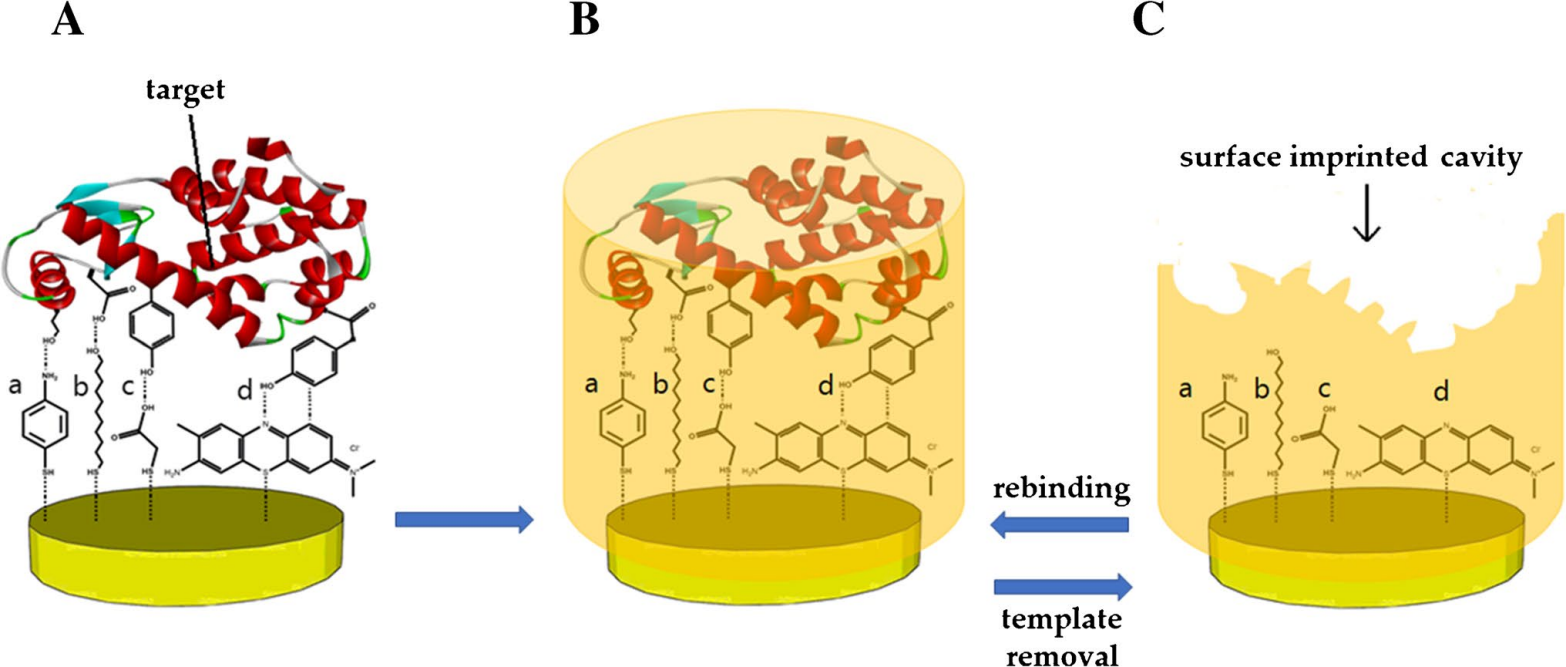
Schematic of surface imprinting coupled with template anchoring to the electrode surface. (**A**) surface immobilization of the protein by SAM-forming units on gold electrode followed by (**B**) the assembly of a polymer shell (orange) around the template and (**C**) the removal of the template to produce the imprinted cavity on the polymer surface. For gold surfaces, common molecular linkers used are (a) 4-aminothiophenol [37, 38], (b) 11-mercapto-1-undecanol [39, 40], (c) thioglycolic acid [41, 42], (d) toluidine blue [43]. The protein structure depicted is lysozyme, which is stored under the code 4LZM at the Protein Data Bank

A happy marriage has been reported in the literature between surface imprinting strategies and coupling chemistry protocols which enable the anchoring of the preliminary template to the electrode surfaces. In this way, both of the main limitations of traditional imprinting can be eliminated: on one hand, the reduced MIP film thickness provided by surface strategies helps to overcome limited protein diffusion, and on the other hand, the anchoring of the template to the transducer surface, if done in an oriented way, can be beneficial in terms of generating uniformly accessible binding sites, limiting drawbacks related to protein orientation/conformation. This process can be achieved through the use of anchoring agents that exploit terminal moieties with affinity to both the electrode surface and the template [36]. A very common material for surface imprinting in MIP sensor development is gold, due to the possibility for spontaneous self-assembled monolayer (SAM) formation through thiol moieties. Other motifs to bind the protein are then exposed and can be used as a linker for the target, leading to simpler surface modification protocols and more straightforward manufacturing of highly sensitive sensing platforms [36]. As a matter of fact, it is widely known that anchoring agents bearing hydroxyl, thiol, and amino motifs allow the spontaneous formation of monolayers of conserved geometric orientation that favor biosensor design. This process on a gold electrode is schematically reported in Fig. 2, illustrating commonly used linkers for the gold surface as well.

**Fig. 3:**
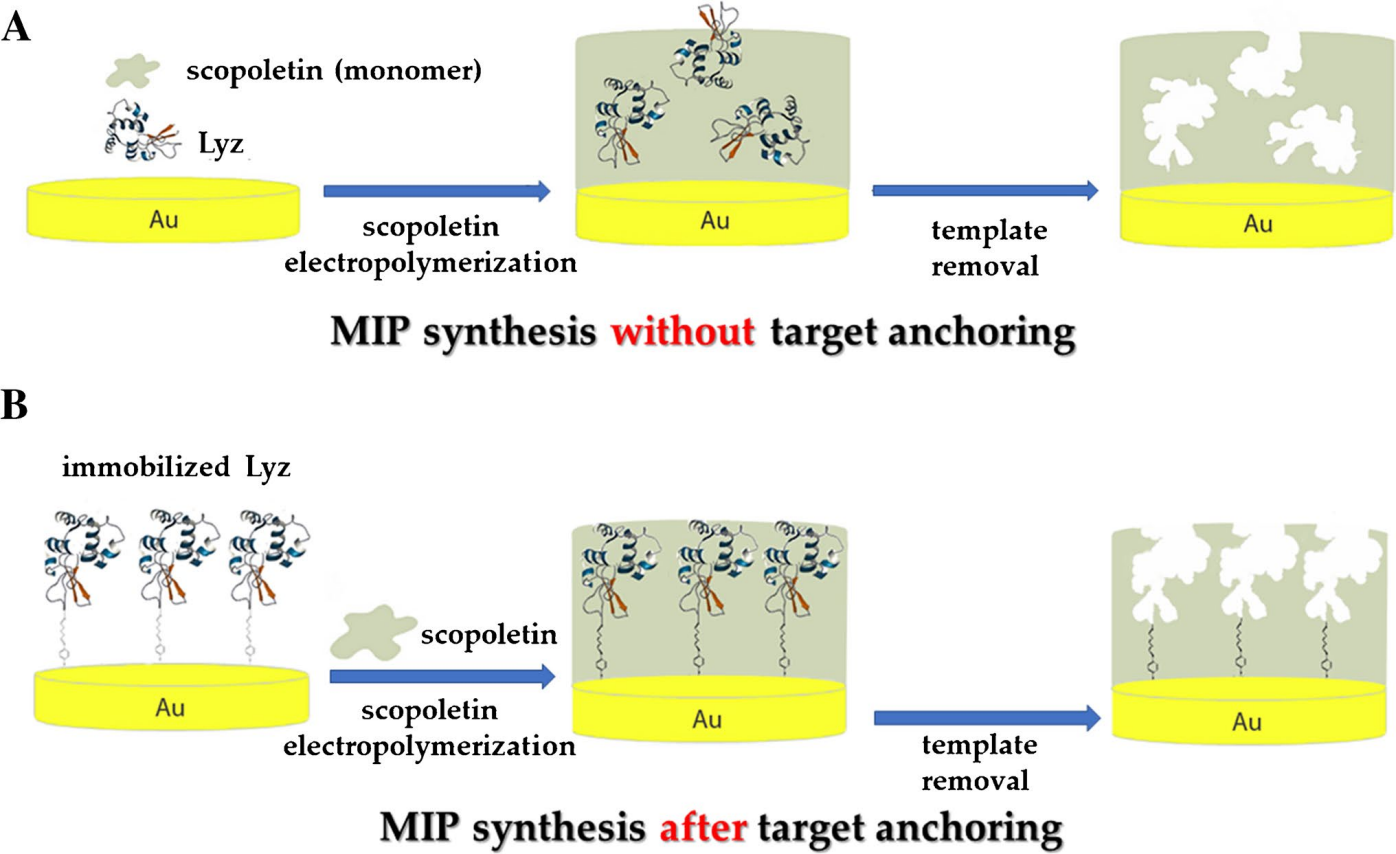
Comparison between two imprinting approaches. (**A**) MIP synthesis by electropolymerization of a solution containing monomer and template; (**B**) MIP synthesis after preliminary anchoring of the target on the electrode surface. Adapted from [37]

The actual advantages from such a two-step approach have been discussed and demonstrated by several researchers [44, 45]. For instance, in a recent report [37], the authors developed an impedimetric MIP sensor for the electrochemical detection of lysozyme (Lyz) and selected the optimal conditions after comparing the analytical performance of sensors obtained by two different imprinting approaches (Fig. 3), namely (i) electropolymerization of a solution containing monomer and template, and (ii) target immobilization on the electrode surface prior the electropolymerization of a monomer solution. The results showed that surface functionalization with the template before MIP assembly led to a threefold increase in the imprinting factor, thereby leading to more reliable quantification. The final sensor showed a linear response over a wide concentration range (150 nM to 20 μM) and a limit of detection (LOD) of 60 nM. Moreover, the sensor prepared according to approach (ii) showed enhanced selectivity with very low interfering values (from 0.07 to 0.24) for each tested molecule (human hemoglobin (HHb), cytochrome C (CytC), bovine serum albumin (BSA), and glucose oxidase).

Interesting results were reported in a very recent work [21] exploiting the facile anchoring of SARS-CoV-2 nucleoprotein onto a gold electrode through a 4-aminothiophenol SAM. After the target immobilization, an m-phenylenediamine-based MIP was electropolymerized on the electrode surface. The developed sensor was able to reach a very low LOD of 15 fM. Nonetheless, the authors did not report any experimental details on the preparation of protein solutions at such a low concentration. The selectivity of the sensor was explored by evaluating its ability to discriminate between target and interfering proteins including a subunit of SARS-Cov-2 spike protein, hepatitis C virus surface viral antigen, cluster of differentiation 48 protein CD48, and BSA. The selection of these proteins was based on the size, isoelectric point, molecular weight, and possible presence in real samples. A considerably higher sensor response was observed against the target protein than the interfering proteins, demonstrating the appreciable selectivity of the fabricated device and promising performance in clinical samples. Moreover, the ability of the sensor to discriminate between the template and CD48, the protein with smaller size and closer isoelectric point, provided additional evidence that the imprinted cavities were complementary to the target protein not only in size but also in arrangement of the functional groups.

**Fig. 4:**
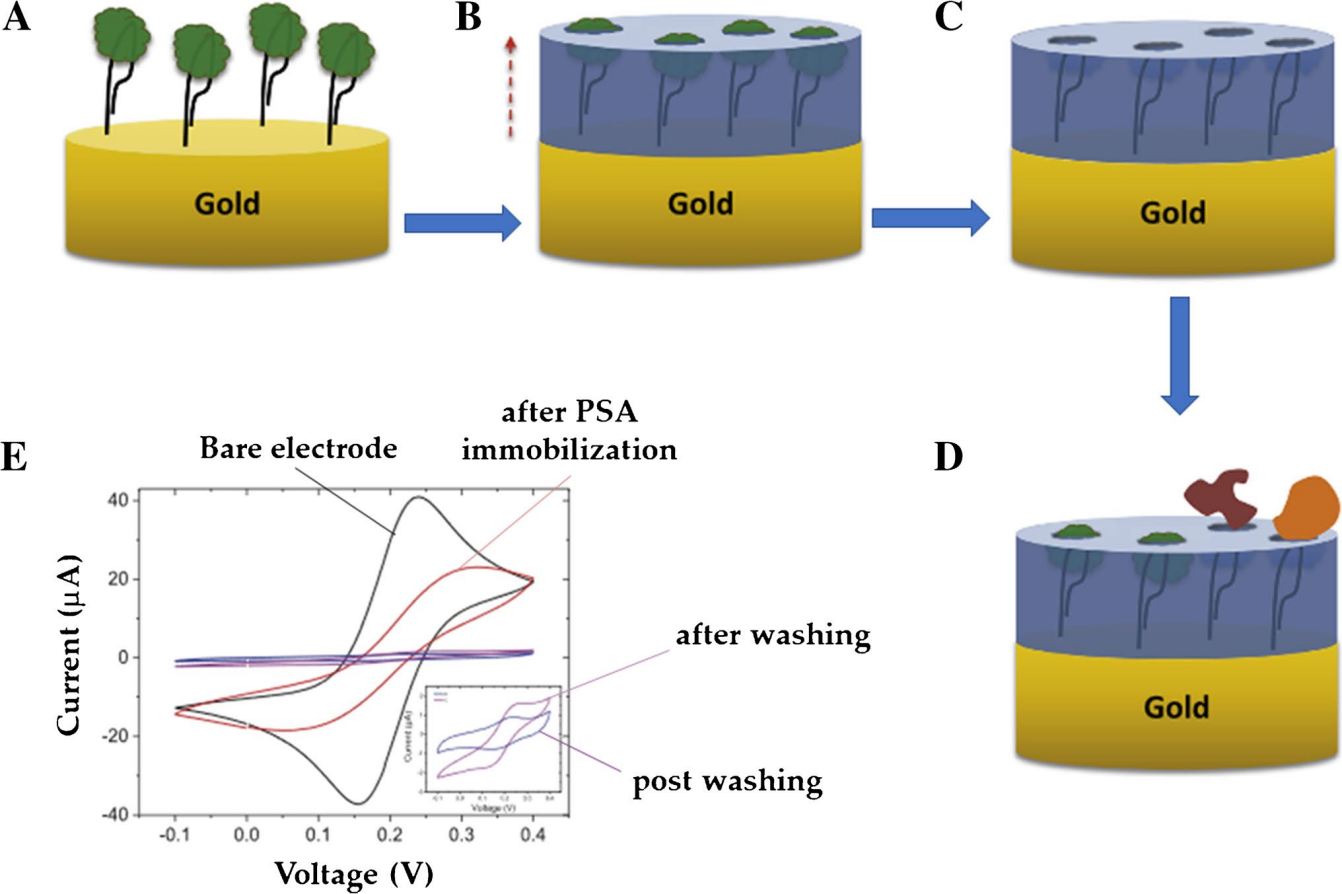
Schematic representation of the synthesis of a hybrid aptamer–MIP sensing material: (**A**) anchoring of the protein–aptamer complex on the electrode surface; (**B**) electropolymerization of a polydopamine film; (**C**) formation of imprinted cavities; (**D**) PSA recognition by sensitive material; **E**) electrode modifications monitored by cyclic voltammetry. Adapted from [46]

Another recent effort described the quick and easy functionalization of gold electrodes with prostate-specific antigen (PSA) (Fig. 4) prior to electropolymerization of dopamine for the synthesis of a hybrid aptamer–MIP formation. In this case, a thiolated DNA aptamer with established affinity for the target was used as linker. The authors reported that the imprinted binding sites and high-affinity aptamers retained on the electrode surface after PSA elution synergistically enhanced the recognition capability of the developed MIP. The resulting electrochemical sensor was very sensitive, with a LOD of 1.0 pg mL^−1^, and showcased a highly ordered topology which contributed to the high sensor selectivity toward the antigenic analyte [46].

The versatile assembly of SAMs on gold electrodes for surface imprinting has also given rise to multi-analyte point-of-care sensors for healthcare applications, such as the concomitant use of PSA and myoglobin (Myo) as surface-imprinted templates for polyacrylamide MIP assembly in a dual-sensing impedimetric sensor, showing detection limits of 5.40 pg mL^−1^ and 0.83 ng mL^−1^, respectively [47].

The use of long-chain thiol-bearing residues has been reported in particular to yield reliable sensors, as evidenced by the use of 11-mercaptoundecanoic in many sensing platforms, as well as the immobilization of double-cysteine-modified peptide nanofilms onto gold surfaces for neuron-specific enolase (NSE) imprinting on polyscopoletin-based MIP, which yielded a detection limit of 0.25 μM [48].

Short-chain SAM-forming units have alternatively been used to anchor already synthesized MIPs on the surface of electrodes. For instance, the anchoring of MIPs on a gold electrode surface by allyl mercaptan was reported for the development of a highly sensitive sensor for the detection of oxytocin [49], which yielded a LOD of 0.0030 ng mL^−1^.

**Fig. 5:**
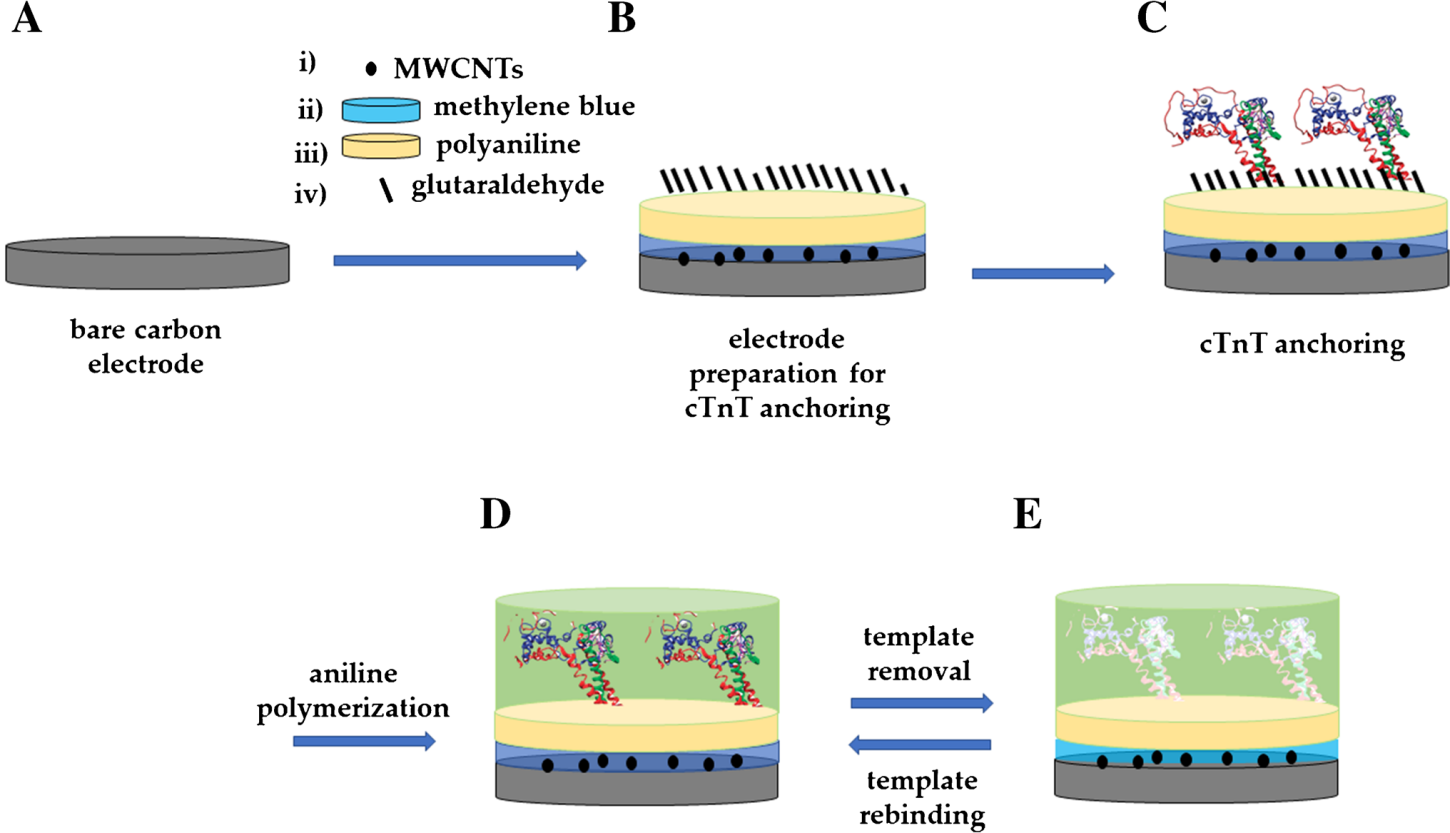
Fabrication of a MIP sensor prepared by surface imprinting and template anchoring to a screen-printed carbon electrode (SPCE). (**A**) The electrode surface was first functionalized with (i) multi-walled carbon nanotubes (MWCNTs) and (ii) methylene blue, and then (iii) a polyaniline layer was electrosynthesized and modified with (iv) glutaraldehyde to obtain (**B**) an anchor layer for cTnT. (**C**) cTnT was anchored on the electrode surface. (**D**) An additional polyaniline layer was electropolymerized around the target. (**E**) After removal of cTnT from the polymer film, surface binding sites able to recognize the target were obtained. Adapted from [55]

Although not providing spontaneous monolayer formation, carbon materials have also been used in the surface imprinting of proteins and other molecules by exploiting π-π stacking interactions between anchoring agents bearing aromatic moieties [50, 51], or by forcing the genesis of anchoring sites through chemical activation [52–54]. For instance, the surface imprinting of cyclic troponin T (cTnT) was reported on carbon-based electrodes [55]. For the sensor development (Fig. 5), screen-printed carbon electrodes (SPCEs) were first functionalized with multi-walled carbon nanotubes (MWCNTs) and then with an electrically deposited methylene blue layer (which was used as the internal electrochemical probe of the sensor). Later, for the actual MIP synthesis, polyaniline was electropolymerized on the as-modified electrode and used as a support layer to bind glutaraldehyde acting as a linker for cTnT. Another aniline-based layer was electrodeposited around the target, which was then removed to obtain the imprinted cavities on the surface of the MIP film. The change in the redox signal of the methylene blue redox probe previously deposited on the electrode was monitored for target determination.

In another work, dengue virus nonstructural protein was surface-imprinted on SPCEs coated with electrospun nanofibers of polysulfone in order to develop MIP-based electrochemical sensors for biomedical applications, which yielded a LOD of 0.30 ng mL^−1^ for the target virus protein [56]. As far as selectivity studies, reported results are not particularly striking. Sensor selectivity was tested only against Lyz and fetal bovine serum. From the reported bar graph, interference ratios of about 0.15–0.2 were obtained, but it is not clear why such interferences were selected. In particular, fetal bovine serum is not a fully defined media component, and as such varies in composition between batches.

Overall, from the reported literature applications, it is apparent that the use of surface imprinting technology in protein electroanalysis is conditioned by the intrinsic features of the electrode surface, as well as those of the anchoring agent and the template. Therefore, the appropriate protocol must take into consideration the surface material and the chemical structure and topology of the anchoring agent, as well as their interaction with the template [16]. A more rational imprinting approach has thus to be designed in comparison with the use of monomer-template mixture, but related advantages certainly justify greater efforts required for this step.

### Epitope imprinting

**Fig. 6:**
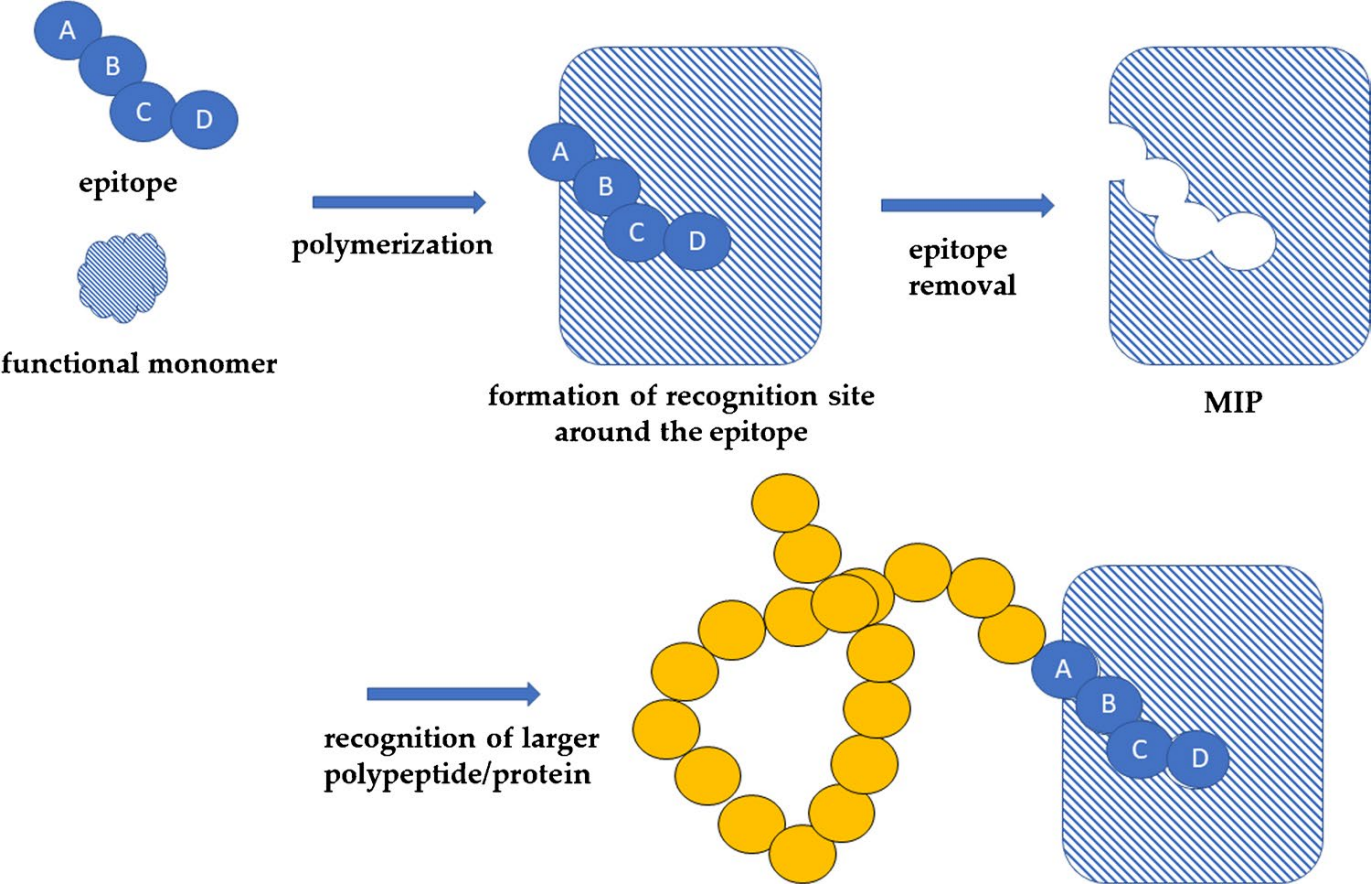
Representation of a classic example of the epitope approach: a small epitope is used to produce imprinted cavities in a MIP matrix capable of selectively recognizing a macromolecule. Adapted from [59]

Epitope imprinting has opened a new frontier in the field of molecular imprinting. This approach consists essentially in using small peptides, rather than entire protein targets, for the synthesis of MIPs. The peptide is generally chosen considering the amino acid sequences that the protein target displays in its outermost portions, which are those most likely involved in interaction with the sensitive MIP membrane [57, 58]. Thus, by imprinting the small characteristic peptide, the imprinted binding sites interact only with small portions of the protein target, hence bypassing the imprinting hindrances of whole proteins attributed to size and conformational flexibility (Fig. 6).

Due to their shorter length compared to whole proteins, epitopes have a predictable primary and/or secondary conformation, in contrast to the complex tertiary structure of whole proteins, whose recognition sites may not be adequately exposed for molecular imprinting applications [57]. An imprinting process of protein recognition motifs can lead to a greater standardization of the imprinted cavity topology in order to better tune the selectivity of the sensor [8, 60].

Epitope imprinting is a relatively recent approach, successfully introduced by Rachkov and Minoura in the 2000s [59, 61]. The authors used this methodology for the development of MIPs for protein separation and analysis. They coined the terminology “epitope approach,” which has since then been used by researchers and scientists working in the field of molecular imprinting to refer to this type of approach, the use of which has increased exponentially over the last 20 years, as reported in several outstanding reviews recently published on the topic [8, 10, 57, 58, 62].

Since the small epitope replaces the macromolecular target during the imprinting process, a preliminary study step is necessary to choose the best epitope candidate starting from the structure of its macromolecular source. There are several alternatives in epitope template selection [10, 62].

- *Terminal sequences of protein targets*: Small linear peptides such as C- and N-terminal portions are used. However, the use of C-terminal portions is much more frequent, probably because post-translational modifications are less frequent for this type of moiety, so that higher correspondence with the terminal portions of the actual target can be expected [61, 63]. The advantages of this approach include the easy and cheap production of linear peptides, the quick selection of binding sites considering only the amino acid sequence of the protein, and the possibility of modifying binding sites with a selected amino acid (e.g. histidine, cysteine residues) to facilitate a specific desired bioconjugation process [64, 65].
- *Peptide sequences extracted from protein target*: The selection of peptide sequences is carried out after a scan of the structure of the protein target, taking care to verify that these portions have the suitable requirements for the experimental conditions of synthesis (e.g., solvent solubility) [48, 65, 66]. As easily argued, in this case the selection of the peptide sequence can be more difficult, but also makes it possible to widen the spectrum of target proteins to be templated and can improve the performance of the resulting MIP. In some cases, the protein sequence has been scanned alongside other proteins of the same family [67]. Another epitope selection method consists in identifying small sequences common to a target family of peptide molecules, such as neurotransmitters, hormones, or toxins [68, 69]. An advantage of this approach is that it allows the resulting MIP to bind multiple molecules that share the same amino acid sequence.
- *Naturally occurring epitopes*: This consists in the use of portions of proteins for which recognition phenomena by antibodies or cell receptors are known. For example, for the development of a sensor for the detection of the bacterium *Neisseria meningitidis*, a nonapeptide of an external protein of the bacterial membrane was chosen as epitope template [70]. The most fascinating aspect of this approach lies in the possibility of achieving a remarkable imprinting effect, as the interactions involved exploit already established immunogenic regions. On the other hand, using previously verified antigenic determinants may also be unfeasible when the target protein has not yet been sufficiently studied.
- *Nonlinear peptides*: Considering that molecular recognition mechanisms in nature often use interactions with secondary and tertiary structures of proteins, the use of linear peptides may be a reductionist approach. For this reason, the use of cyclic peptides that more closely mimic certain portions of proteins is becoming popular [71, 72]. The selection of suitable peptides in this case necessarily requires the use of bioinformatics tools (see below) for 3D visualization, surface functionality assessment, and secondary structure prediction steps by specialized platforms.
- *Non-peptide-based epitopes*: It is possible to use non-protein molecules, such as mono- and oligosaccharides, for the development of MIPs designed for the recognition of glycoproteins or for the detection of cells carrying such molecules on the surface [10]. In this approach, the selection of epitope is facilitated but its use is limited to the imprinting of a restricted range of proteins bearing specific glycosylation sites.

From all the above, it is evident that the epitope selection could be difficult. In this sense, useful available tools are provided by bioinformatics resources that enable the scouting of epitopes based on the macromolecular target to be revealed, also giving indications about the feasibility of the process of molecular imprinting (monomer–template interaction, in-solvent stability, etc.). It should be highlighted that by computational approaches, the binding energies between template and functional monomer may be simulated to obtain information on potential complex stabilities with the functional monomers. In this regard, an interesting work was recently published by Bossi’s group [62]. In their review, they surveyed a series of reports about MIPs obtained by epitope imprinting, indicating informatics resources freely available online that might offer key tools for the selection of suitable epitopes for the imprinting process. In another work, Busato et al. [73] presented the development of an open-access platform called MIRATE (MIps RATional dEsign Science Gateway), which helps imprinters throughout the entire MIP synthesis process. The user-friendly interface also makes the tool accessible to users not specialized in computational modeling.

Although assisted by computational approaches, the choice of the optimal epitope and the most suitable epitope approach is not trivial and must be accurately performed in relation to the specific protein to imprint. The length of the epitope peptide is a crucial parameter for the imprinting and subsequent recognition process. Peptides that are too small can lead to low-selectivity binding sites. On the other hand, peptide chains that are too long possess a higher degree of flexibility in solution, and intramolecular interactions may occur, forming undesired 3D structures of the template and thus possibly affecting the imprinting process [10]. Moreover, it is known that oligopeptides consisting of up to 30 amino acid units may not be compatible with all polymerization strategies, and many epitope chains are known to undergo aggregation in aqueous environments, which must also be taken into consideration when using these biomaterials in molecular imprinting technologies [8, 57].

Epitopes to be used during the molecular imprinting process can be obtained through their extraction and isolation from their biological source, but also through faster synthetic approaches that produce only the peptide chain required for MIP assembly [8, 74]. Due to its flexibility, epitope imprinting has enabled the development of tailored MIP-based sensors for considerably rare or hard to obtain biomarkers [58], for which the use of the entire molecule in the imprinting process would have the additional weakness of consuming large amount of highly costly molecules.

To understand the high potential of the epitope imprinting approach, as cleverly highlighted in a recent work [62], it has to be taken into consideration that the protein–protein interactions occur via a defined portion of the protein surface, showing that just a selected part of the protein is responsible for the recognition of a molecular partner. Analysis of the protein complexes shows that despite the limited surface involved in the recognition, remarkably strong contacts can be achieved, justifying the very low dissociation constants in the picomolar range that typically characterize the highly selective protein interaction with receptor possessing complementarity toward only a small fragment, i.e., a peptide, of the whole protein.

**Fig. 7:**
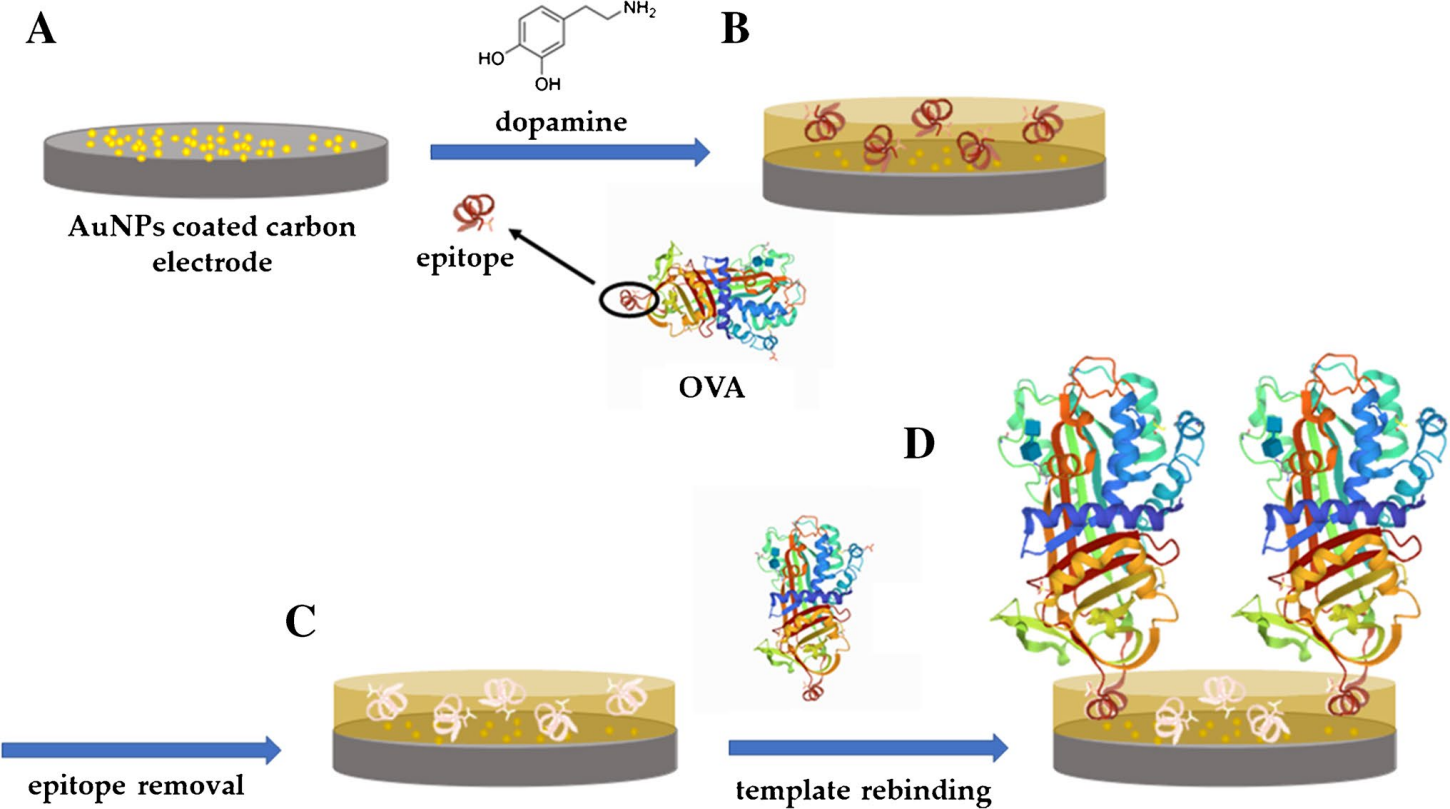
Schematic representation of a MIP for albumin synthesis by the epitope approach: (**A**) SPCE functionalized with AuNPs; (**B**) electropolymerization of a solution containing epitope and dopamine; (**C**) epitope removal from polymer matrix; (**D**) template binding on the MIP surface. Adapted from [76]

Epitope imprinting MIP-based sensors for proteins and macromolecules have been reported in several fields, ranging from healthcare to foodstuff quality control [17, 22, 60, 75]. For instance, in a very recent report [76], it was used for the imprinting of ovalbumin (OVA) on SPCEs (Fig. 7).

To this aim, a polymerization mixture containing OVA IgE-binding epitope (amino acid sequence of ovalbumin) as template and dopamine as monomer was used for the electro-synthesis of a polymer layer on a working electrode previously functionalized with gold nanoparticles. The templates were removed by washing the electrode in 1 M NaOH solution for 30 min, followed by distilled water for 15 min, to obtain the final MIP. The resulting sensor was able to detect the target in a linear range from 23.25 to 232.50 nM, with good selectivity against other proteins such as human serum albumin (HSA), BSA, and Lyz. BSA and HSA were selected as naturally globular proteins, similar to OVA, while Lyz and OVA are both present in egg white. Only limited interference from Lyz was observed and was found to be slightly higher than BSA and HSA. According to the authors, this could be due to the difference in the molecular weight of the tested proteins, as Lyz, due to low molecular weight, might diffuse into the recognition cavities and interfere with the electron transfer of the sensing interface.

In another work, Cheng-Jung et al. [63] developed an electrochemical sensor for insulin detection in serum samples. C-terminal polypeptide of insulin, as template molecule, was self-assembled directly on the surface of a gold electrode, before electrosynthesis of the MIP layer using o-phenylenediamine as functional monomer. The authors did not exhaustively explain how the polypeptide adsorbs on the surface of the electrode, but the effectiveness of this step was confirmed indirectly by monitoring the signal change of a redox probe. The final sensor was able to detect insulin at femtomolar levels, with a detection limit of 7× 10^−15^ M. Also, in this case, the authors did not report any experimental details on the preparation of protein solutions at such a low concentration.

**Fig. 8:**
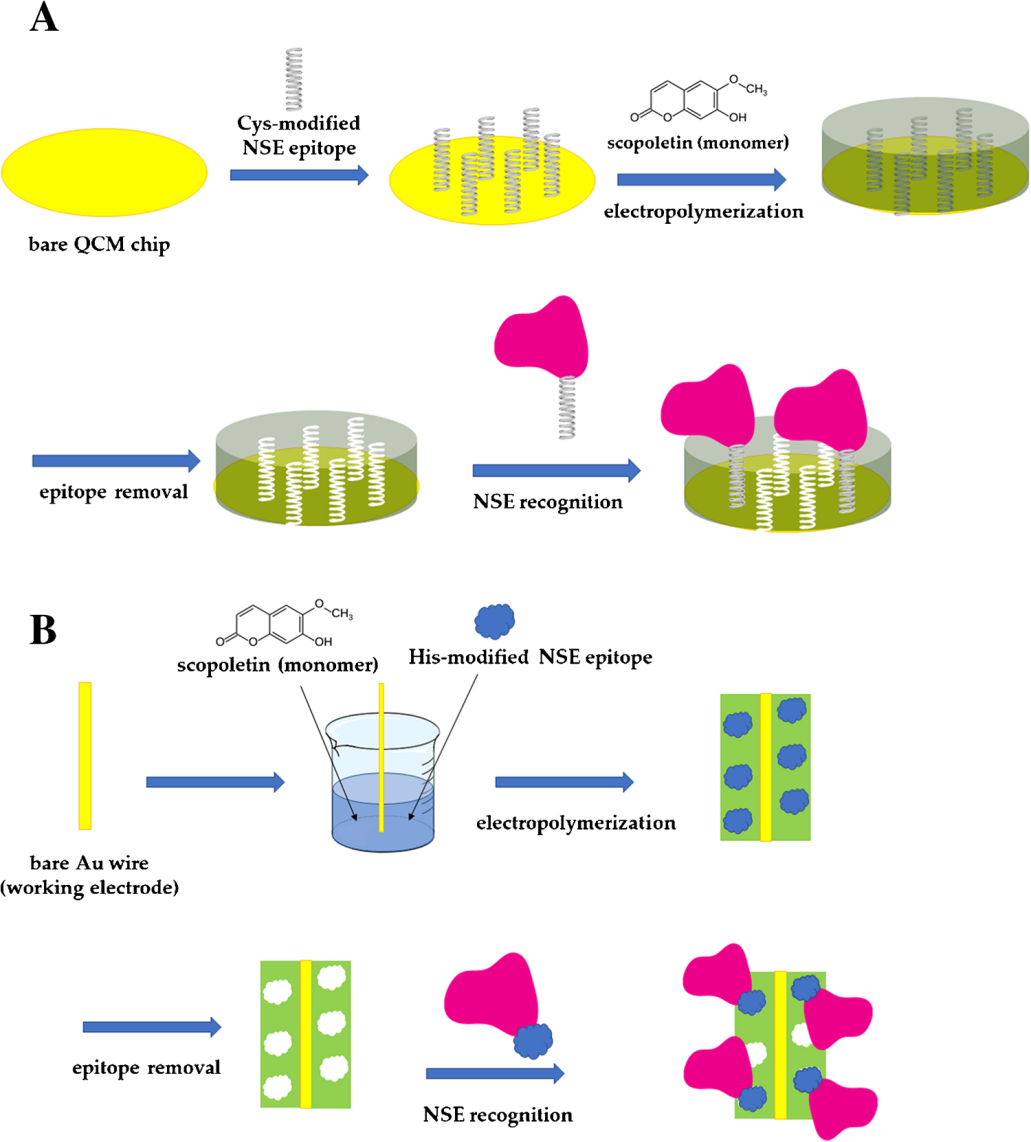
Two different ways to perform the epitope approach to obtain electrochemical MIP-based sensors for NSE. (**A**) Epitope (cysteine-modified epitope) immobilization on the electrode surface prior to the electrosynthesis of a polymer (from scopoletin monomer). Subsequent removal of the epitope produces the MIP. (**B**) Electrochemical polymerization of a solution containing epitope (histidine-modified epitope) and monomer (scopoletin). All functionalization steps were monitored by checking the change in the electrochemical signal of the [Fe(CN)_6_]^3−/4-^ probe. Adapted from [65]

As discussed with regard to the imprinting of the whole protein, epitope imprinting can also be basically performed from the polymerization of a solution containing the selected epitope and monomer, and preliminarily anchoring of the epitope on the transducer surface prior to polymer synthesis through direct chemisorption or the use of an anchoring agent such as SAM-forming units [60]. The combination of epitope and surface imprinting strategies is very common [65]. An interesting work was published by Tchinda and coworkers [65], who compared the performance of two sensors for the detection of NSE biomarkers, obtained with and without the anchoring of the epitope to the surface of gold electrodes and on quartz crystal microbalance (QCM) chips (Fig. 8). In this case, the two approaches were revealed to be similarly effective in preparing highly selective MIPs.

The above-reported results, although not pretending to exhaustively illustrate the remarkably wide and ever-increasing field of epitope imprinting, give an idea of the great potential of this approach, which is rapidly expanding and changing the nature of macromolecule imprinting, making it more practicable and closer to a multitude of applications thanks to eliminating the need to use the whole protein, with related issues of high costs and possible above-discussed limitations in imprinting efficiency.

## MIP format suitable for the electrochemical sensing of macromolecules

To overcome the abovementioned issues related to macromolecule imprinting, researchers have proposed not only the development of surface-based and epitope imprinting procedures, but also the employment of nanosized MIPs with surface-exposed imprinted sites due to their high surface-to-volume ratio. This facilitates MIP–protein interaction and promotes analyte diffusion to the electrode surface, which is particularly crucial for obtaining a readable signal in electrochemical platforms [77]. Therefore, two major approaches have been proposed for this purpose:

- employing thin and ultrathin MIP films; and
- using MIPs in the form of nanoparticles (MIP NPs).

Once one or the other of these MIP formats has been chosen, the age-old question of how to integrate MIPs with electrochemical sensors must be addressed. There are various approaches used for this purpose, and the target detection technique also varies accordingly [7, 78]. While in the case of MIP NPs, their synthesis and integration with the transducer have to be decoupled, when dealing with MIP film, two strategies for their integration with the electrochemical sensing platforms can be used [7, 13, 78]: (i) in situ polymerization, with direct synthesis of the MIPs onto the transducer, and (ii) ex situ polymerization, by incorporation or immobilization of previously prepared MIPs (Table 1). As easily argued, the first approach allows us to easily achieve MIP synthesis and integration with the sensors in a single step. The second strategy, on the other hand, allows us to complete MIP synthesis and characterization before their immobilization on the transducer, so both steps can be independently optimized.

**Table 1:** Examples of MIP integration with the transducer surface in electrochemical sensors for target protein detection

Integration approach	MIP format	Monomer	Polymerization methods ^1^	Electrochemical readout ^2^	Analyte	Rebinding/response time (s)	Linear range	LOD	Ref.
	MIP film membrane on carbon nanotubes/graphene composite modified carbon electrode	Aniline	Electropolymerization (CV)	DPV	BSA	300	100 pg mL^−1^ to 100 μg mL^−1^	60 pg mL^−1^	[79]
In situ synthesis	MIP film	Scopoletin	Electropolymerization (MAT)	EIS^1^	Lyz	3600	150 nM to 20 μM	60 nM	[37]
	MIP film	Diethyl-aminoethyl methacrylate (DEAEM)	Controlled/living radical polymerization (CLRP)	DPV	Brain-derived neurotrophic factor (BDNF)	1800	0.01–0.06 ng mL^−1^	6 pg mL^−1^	[31]
	MIP nanofilm	Scopoletin	Electropolymerization (CV)	CV	(a) Human serum albumin (HSA) (b) Ferritin (Fer)	2100	(a) 20–100 mg L^−1^ b) 40–360 mg L^−1^	(a)4 mg L^−1^ (56 nM) (b)11 mg L^−1^ (10 nM)	[80]
	MIP layer	11-mercapto-1-undecanol	SAM deposition	Potentiometry	(a) Myoglobin (Myo) from equine (b) Bovine hemoglobin (BHb)	600	(a) 0–250 μg mL^−1^ (b) 0–300 μg mL^−1^	n.d.	[81]
	MIP layer	11-Mercapto-1-undecanol	SAM deposition	CV EIS Potentiometry	Carcinoembryonic antigen (CEA)	300	0–8.5 ng mL^−1^	0.5 ng mL^−1^	[82]
	MIP composite membrane onto chitosan-coated magnetic nanoparticles (MNPs)	Pyrrole	Electropolymerization (CV)	DPV	BSA	30	10^−4^ to 10^−10^ g mL^−1^	3 × 10^−11^ g mL^−1^	[83]
	MIP film	Itaconic acid	Controlled/living radical polymerization (CLRP)	SWV DPV	PSA	600	10 pg L^−1^to 60 mg L^−1^	0.25 pg mL^−1^	[84]
	MIP thin film	Pyrrole	Electropolymerization (CV)	SWV	Carbohydrate antigen 125 (CA-125)	900	0.01 to 500 U mL^−1^	0.01 U mL^−1^	[85]
	MIP film	2-aminophenol	Electropolymerization (CV)	EIS	Myo	300	4-53 μg mL^−1^	3.5 μg mL^−1^	[86]
	MIP layer	(a) scopoletin (b) o-PD	(a) Electropolymerization (MAT) (b) Electropolymerization (CV)	(a) AMP (b) DPV	Tyrosinase	7200	(a) n.d. (b) 0–50 nM	4 nM	[87]
	MIP nanocomposite layer with gold-decorated graphene nanosheets	Dopamine	Electropolymerization (CV)	DPV	PSA	600	1 pg mL^−1^ to 100 ng mL^−1^	0.15 pg mL^−1^	[88]
	MIP film	m-Phenylenediamine (m-PD)	Electropolymerization (CV)	DPV	SARS-CoV-2 antigen	900	2–110 fM	15 fM	[21]
	MIP film	(a) 4-bis(2,2′-bithien-5-yl)methylbenzoic acid glycol (b) 2,4,5,2′,4′,5′-hexa(thiophen-2-yl)-3,3′-bithiophene	Electropolymerization (CV)	Capacitance	Oxytocin	n.d.	0.06–1 mM	60 μM	[89]
Ex situ synthesis	MIP nanoparticles	Chitosan (CS)	Photopolymerization	DPV	BSA	300	10^−14^ to 10^−9^ mg mL^−1^	n.d.	[90]
	MIP MNPs	(a) *N*,*N*′-methylene-dipropylene amide (MBA) (b) Methacrylic acid (MAA); (c) Acrylic amide (AAm); (d) *N*-isopropylacrylamide(NIPAAm)	Chemical polymerization (by initiator agent)	CV DPV	Human immunoglobulin G (IgG)	480	0.1–50 ng mL^−1^	0.02 ng mL^−1^	[91]
	MIP MNPs	(a) NIPAAm (b) AAm (c) MBA	Chemical polymerization (by initiator agent)	DPV EIS	Lyz	660	0.05–0.8 μg mL^−1^	1.6 × 10^−3^ μg mL^−1^	[92]
	MIP cryogel	(a) acrylamide; (b) *N*,*N*-methylenebisacrylamide; (c) acrylic acid; (d) diallylamine	Cryogenic polymerization	EIS	BSA	3600	10^−16^ to 10^−12^ M	7 × 10^−18^ M	[93]
	MIP hydrogel film	(a) NiPAAm (b) AAm (c) methylene-*N*,*N*-bis(acrylamide) (MBAAm)	Bulk polymerization	CV EIS DPV	BSA	1200	0.02–10 uM	0.012 μM	[94]

Abbreviations: MAT: multi-step amperometry technique; CV: cyclic voltammetry. EIS: electrochemical impedance spectroscopy; DPV: differential pulse voltammetry; SWV: square wave voltammetry; AMP: amperometry

### MIP film

Currently, several approaches are available to synthesize MIPs in the form of film on the transducer surface, with the possibility for an electrochemical readout of the signal related to the molecular recognition event. The main strategies for MIP film synthesis are as follows:

- drop-casting or spin- and dip-coating of previously synthesized polymers on the electrode surface [94–98] or of solutions containing template and monomers which are subsequently polymerized in situ [30, 95];
- use of thiol derivatives to form self-assembled layers with molecular recognition properties on gold surfaces [81, 82];
- synthesis of films by grafting polymerizable groups and/or initiators onto the support surface [99–102];
- electropolymerization of a mixture of electroactive monomers and targets [103] or, alternatively, electrosynthesis of a polymer film after the immobilization of the target on the electrode surface [16, 37, 85].

- *Drop-casting or spin- and dip-coating of previously synthesized polymers or solutions containing template and monomers on the electrode surface*

The strategy consisting in the drop-casting of already formed polymer on the electrode surface [95] was recently exploited in a work by Wei et al. [94], who developed an electrochemical sensor capable of detecting BSA in a linear range between 0.02 and 10 μM with a LOD of 0.012 μM. To assemble the sensor, a pre-imprinted hydrogel was first synthesized by free radical polymerization of a series of monomers in the presence of the template. Later, the pre-imprinted hydrogel solution was deposited on the surface of glassy carbon electrodes by drop-casting, leaving polymerization to proceed for a further 30 min, ensuring adhesion of the sensitive material to the electrode surface. A similar approach was used in another work [96], where an electrochemical sensor was developed for the detection of bovine hemoglobin (BHb) by placing a thin layer of preformed MIP directly on the surface of a glassy carbon electrode by drop-casting. The MIP gel was held in place by a system of membranes attached to the electrode with the aid of a rubber o’ring. With this configuration, the authors claimed to be able to determine the target through its own redox activity.

**Fig. 9:**
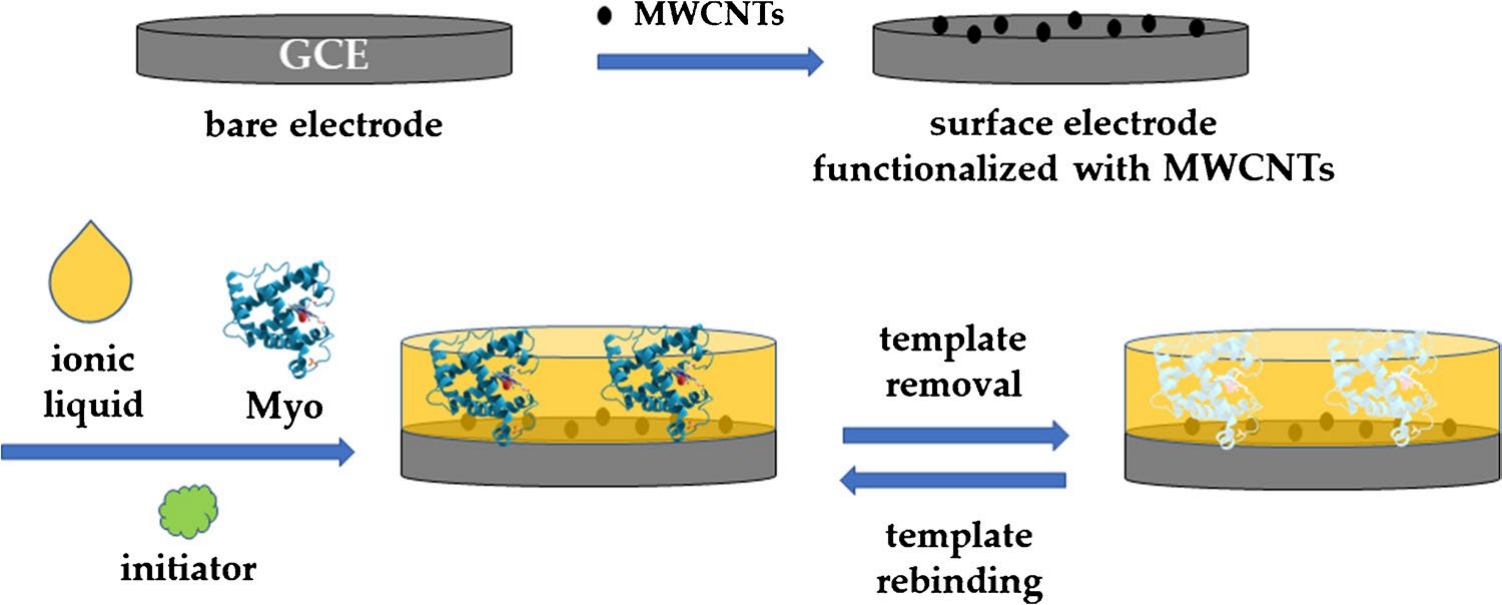
Functionalization scheme of a glassy carbon electrode to obtain a MIP sensor for myoglobin, prepared by in situ polymerization of a solution containing ionic liquid, template, initiator, and electrolyte. Prior to in situ MIP synthesis, the electrode is functionalized with MWCNTs. Adapted from [30]

In other cases, a solution containing monomers and template is directly drop-cast onto the electrode surface followed by polymerization, creating sensitive layers capable of selectively recognizing the target. A multitude of MIPs have also been produced in this manner [95], although only a few examples are focused on protein detection, as in the case of the work by Wang and coworkers [30], who fabricated a MIP-based electrochemical sensor for the detection of Myo (Fig. 9), a heme protein with oxygen-binding properties, used as a biomarker for the diagnosis of acute myocardial infection. A MIP layer was obtained by in situ free radical polymerization, after drop-casting of a solution containing monomer, initiator, ionic liquid (IL), and target on the surface of glassy carbon electrodes, previously modified with MWCNTs, to enhance the conductivity of the working electrode. The optimized sensor was able to detect the protein in a wide range (60.0 nM to 6.0 μM) with a low detection limit (9.7 nM). The authors also applied this electrochemical sensor to determine Myo amount in spiked plasma, and a recovery of 96.5% was reported. To evaluate the selectivity, eight potential interferents, namely BHb, BSA, CytC, OVA, ascorbic acid, glycine, L-cysteine, and L-histidine, were measured independently. Although it was found that the variation in current due to Myo was higher than that with other analytes, a non-negligible response was observed for BHb. According to the authors, this was because both Myo and BHb contain an organic prosthetic group for binding oxygen, along with other similarities in the sequence of the alpha and beta chains. Although the authors concluded that the sensor was able to discriminate BHb and Myo, some doubts remain considering that the sensor response to Myo was only twofold in comparison with BHb.

It is interesting to highlight that the authors here used an IL with an amino group and a vinyl group (1-{3-[(2-aminoethyl)amino]propyl}-3-vinylimidazole bromide) as a functional monomer. The advantage in such a choice lies in the IL properties including high thermal stability, good conductivity, excellent water solubility, and biocompatibility. In addition, as with many ILs, it can be polymerized at room temperature, avoiding any damage to the protein molecules. The use of ILs as both a monomer and a solvent in the synthesis of MIPs for macromolecules is still largely unexplored. It should be advanced as being highly beneficial for moving toward a green and sustainable imprinting technology, reducing the use of organic solvents and promoting eco-friendly conditions [104].

(b) *Thiol derivatives to form self-assembled layers with molecular recognition properties on gold surfaces*

**Fig. 10:**
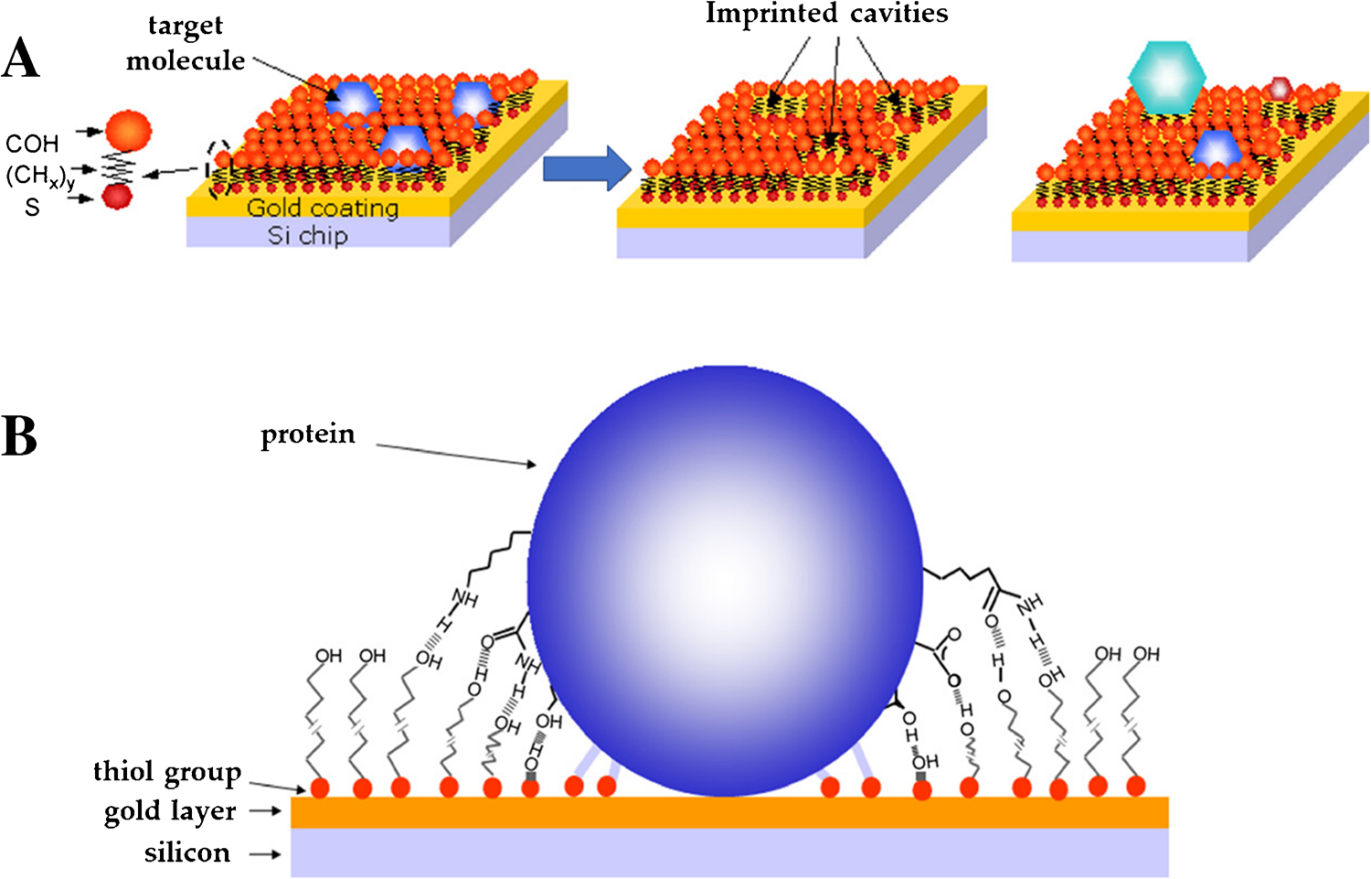
(**A**) Preparation scheme of a SAM layer with molecular recognition capabilities; (**B**) possible interactions between target protein and SAM layer. Adapted from [81]

Other researchers [81, 82] have proposed drop-casting of thiol solutions containing the template for the formation of SAM incorporating the protein targets on gold working electrodes. As in the case of the polymerization process, subsequent removal of the target would lead to the formation of imprinted cavities and thus a MIP layer (Fig. 10).

This strategy was proposed by Yu et al. [82], who developed a potentiometric sensor for the detection of carcinoembryonic antigen (CEA), a biomarker of different cancer types including pancreatic, breast, and colon cancer. The authors used a thiol compound (11-mercapto-1-undecanol) solution, containing CEA, to produce a hydrophilic SAM on the working electrode, which demonstrated the ability to recognize the target after its removal. Although such a system is extremely simple to be designed and implemented, its performance could suffer from poor selectivity, especially in the case of interfering molecules structurally similar to the target [7].

(iii) Synthesis of films by grafting polymerizable groups and/or initiators onto the electrode surface

In situ MIP deposition can also be achieved by surface-confined polymerization upon grafting of polymerizable groups or initiators on the transducer surface [4, 29, 82]. In some cases, this “grafting from” approach has been successfully combined with controlled/“living” radical polymerization (CLRP) techniques [105, 106], which is a family of synthetic strategies whose common feature is to limit and control the number of radicals that react to form the polymer, contrarily to free radical polymerization, which can make it difficult to prepare polymeric layers with well-defined thickness. Briefly, in CLRP the monomer is added to the active chain end and not to another monomer, so that as polymerization continues, monomers will continue to be added to the growing chain [107]. CLRPs permit chain growth to be controlled by controlling the growth and termination steps, acting on the conditions of synthesis (initiators, monomers, modulation of reaction initiation by light, heat, etc.). The combination of the “grafting from” approach with CLRP methods has proven to be a versatile strategy for fine-tuning the growth of the polymer layer, exploiting light- [25, 31], heat- [4], or electrochemically [32] mediated polymerization, and allowing extreme control of the molecular weight and thus the thickness of the resulting polymer [108]. This is due to the excellent possibility for easy suspension and reactivation of the synthesis process (by acting on the trigger factor), increasing the opportunities for better optimization of the final MIP [7, 108].

**Fig. 11:**
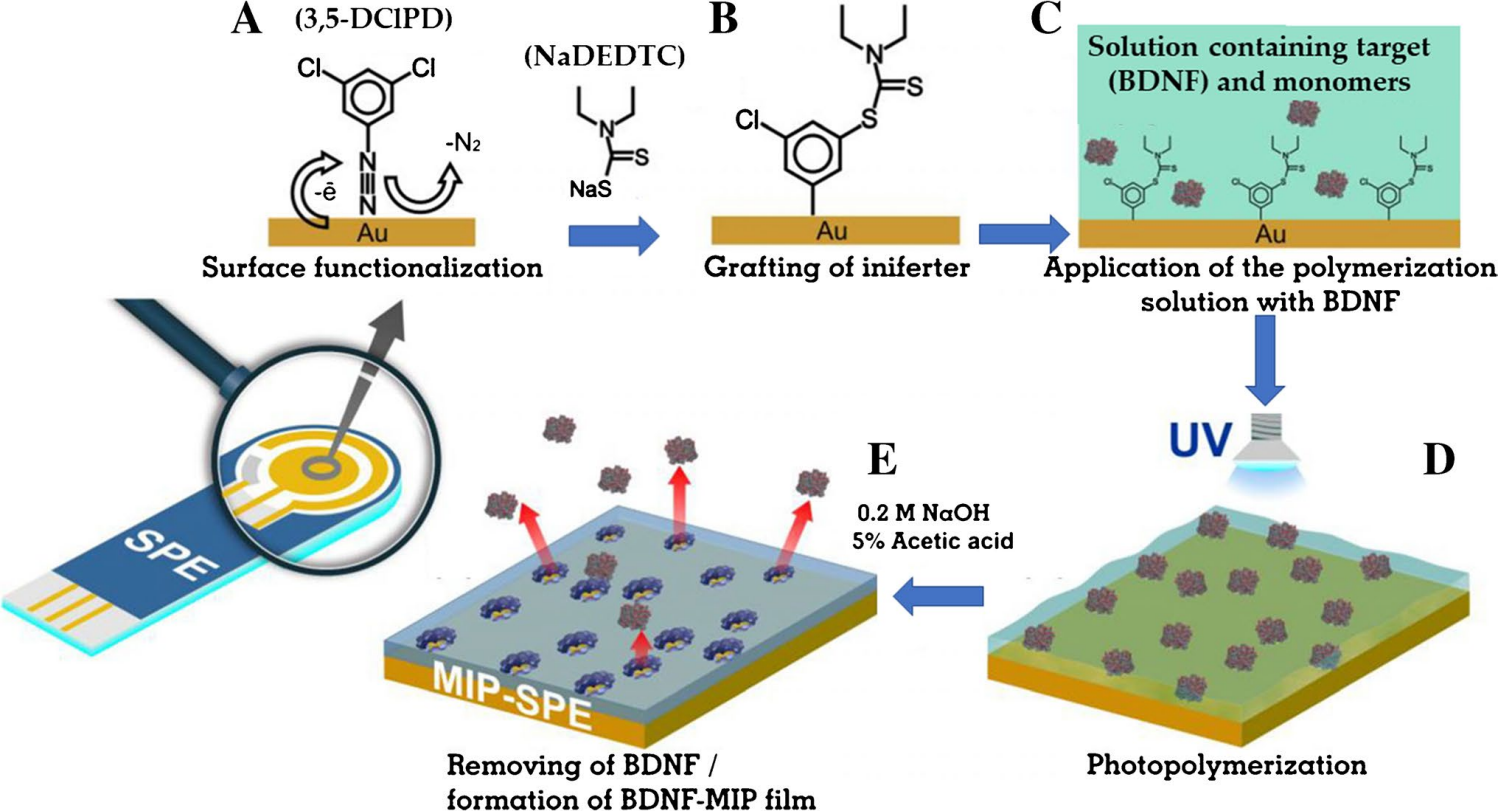
Preparation scheme of a MIP-based electrochemical sensor for BDNF protein by grafting the iniferter before starting photopolymerization. (**A**) Functionalization of SPE with 3,5-DClPD (a diazonium salt); (**B**) grafting of the iniferter (sodium diethyldithiocarbamate, Na-DEDTC) to 3,5-DClPD; (**C**) coating of the SPE with the solution containing a mixture of the functional (DEAEM) and cross-linking (BAA) monomers and the target protein (BDNF); (**D**) photopolymerization of the monomers under UV irradiation; (**E**) removing BDNF from the polymer to form BDNF-MIP on the surface of the SPE. Adapted from [31]

An example was reported by Kidakova et al. [31] (Fig. 11). The authors developed an electrochemical sensor for a clinically relevant protein, brain-derived neurotrophic factor (BDNF). The MIP film on the surface of a screen-printed gold electrode (SPGE) was synthesized by surface-initiated CLRP. In short, an initiator, in particular an iniferter (initiator–transfer agent–terminator) agent, was attached to a sensor surface and then the modified electrode was exposed to a solution containing a mixture of functional monomer, cross-linker, and target protein (see Table 1 for details). The photopolymerization was carried out by UV irradiation. The sensor was able to detect the target in a range of 0.01–0.06 ng mL^−1^ , with an interesting detection limit equal to 6 pg mL^−1^. Unfortunately, the selectivity was not particularly satisfactory, as the sensor showed a significant response to each tested interference, especially at low concentration (0.02 ng mL^−1^). Although the authors highlight sensor selectivity enhancement at higher concentration (0.06 ng mL^−1^), only an approximately twofold increase in comparison to interferences was observed.

**Fig. 12:**
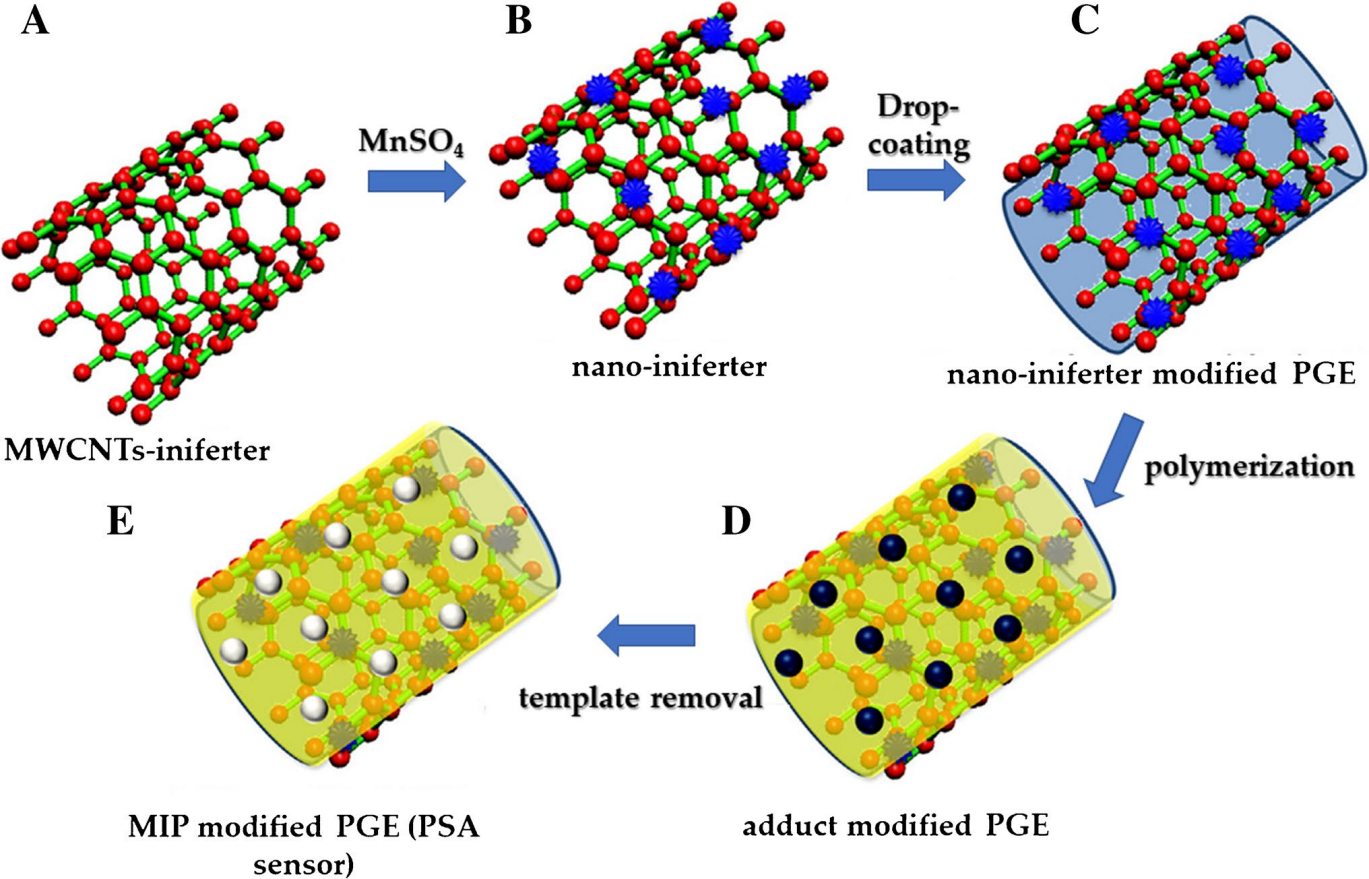
Synthesis scheme of a MIP for PSA protein: MWCNTs were firstly functionalized with Mn nanoparticles to produce a nano-iniferter. This material was used to functionalize a pencil graphite electrode (PGE) by drop-coating. Later, the MIP was obtained on the surface of the working electrode by thermally induced polymerization. Adapted from [84]

In another work [84], Patra et al. developed an electrochemical sensor for the detection of PSA by combining the use of iniferter-induced radical polymerization and MWCNTs, which were firstly functionalized with dithiocarbamate groups and then decorated with Mn nanoparticles to produce a nano-iniferter (Fig. 12). The as-assembled nanomaterial was then anchored to the surface of a pencil graphite electrode (PGE) and used to initiate the surface imprinting of PSA with itaconic acid as the functional monomer and ethylene glycol dimethacrylate (EGDMA) as cross-linker in dimethyl sulfoxide at 50 °C. The final sensor was able to detect PSA by square wave and differential pulse voltammetry (SWV and DPV), showing a detection limit as low as 0.25 pg L^−1^.

An original application of such an approach was proposed by Sun and coworkers [32]. In this work, MIP was prepared on the surface of a Au electrode by electrochemically mediated atom transfer radical polymerization (eATRP), with hemoglobin (Hb) acting as both catalyst and template molecule. To this aim, the electrode surface was first functionalized with an initiator and then exposed to a solution of monomer, cross-linker, and target. Chrono-amperometry was then performed, applying a potential corresponding to Hb reduction, in order to exploit the reduced Hb-Fe(II) form as a catalyst of the polymerization reaction. The as-assembled voltammetric sensor showed a linear response for the target within a logarithmic concentration range from 10^−10^ to 10 mg L^−1^.

As an alternative to the above, rather than anchoring initiator agents on the electrode, it is possible to graft compounds with polymerizable moieties to the transducer surface [29].

It can be highlighted that, although the use of a “grafting from” approach combined with the CLRP method is steadily increasing [109, 110], the number of works exploiting it for the development of MIP-based electrochemical sensors for proteins and macromolecules still does not reflect their potential.

(iv) *MIP electropolymerization*

When exploring the panorama of MIP-based electrochemical sensors, it easily emerges that electropolymerization has been mostly used as a successful approach for integrating the MIP layer with the transducer surface. Since its first applications in the imprinting of small molecules [111–113], benefits related to such an approach have also been widely recognized with macromolecules as template [4]. One of the most fascinating aspects of electrochemical polymerization, which justifies its large use in sensing schemes, is the possibility for easy integration of the MIP layer with the transducer surface, even in the case of substrates with unconventional geometry [114, 115], affording a low-cost and easy-to-use setup. The resulting MIP thickness, which plays a vital role in both template removal and rebinding steps [24, 35, 116], can be easily controlled by simply monitoring the amount of circulated charge during the electropolymerization process and, related to this, the scan rate, specifically when electropolymerization is carried out by cyclic voltammetry [14, 117]. Other properties of the resulting MIP possibly critically influencing the imprinting process can be tuned by varying different electropolymerization experimental conditions, as in the case of polymer porosity, which can be modified by the supporting electrolyte properties [118]. When considering in particular macromolecule imprinting, some additional benefits have to be pointed out. Starting from water-soluble electropolymerizable monomers [4], and requiring the application of water-compatible potential windows [119], MIP deposition can easily take place in an aqueous environment, thus preserving protein stability and limiting the risks related to protein denaturation. Moreover, the use of a buffered polymerization solution can increase imprinting efficiency, as a differentially charged state of the protein template can be promoted, thus favoring monomer–template electrostatic interactions [12]. Also, the abovementioned easy tuning of MIP thickness is a key aspect especially in the imprinting of protein, as the issue of hindered template rebinding and extraction can be particularly significant due to the bulky template. As discussed in section 1.1, the precise control over electrosynthesis enabling the fine-tuning of the polymer layer thickness can be conveniently exploited in surface imprinting of protein templates. Another aspect which undoubtedly contributes to extending the electropolymerization strategy from small-molecule to macromolecule imprinting is the availability of a wide range of suitable monomers [4], which determines, in turn, the possibility to control/increase the amount and the type of available functional groups involved in monomer–template interaction. Monomers such as aniline [120, 121], pyrrole [85, 122], o-phenylenediamine [123, 124], 3-aminophenylboronic acid [125, 126], and scopoletin [37, 80, 127] have gained popularity in MIP electrosynthesis and have also been found to be suitable in the imprinting of macromolecules. This is the case, for example, of aniline and aniline-like compounds, such as o-phenylenediamine, which are favored for protein imprinting because they carry functional moieties that allow multiple interactions, including hydrogen bond, π-π bond, and electrostatic forces, with the target molecules [80]. In the case of glycoprotein imprinting, a very suitable monomer is 3-aminophenylboronic acid, which carries the boronic groups capable of forming reversible covalent bonds with diol groups of glycoproteins [128, 129].

**Fig. 13:**
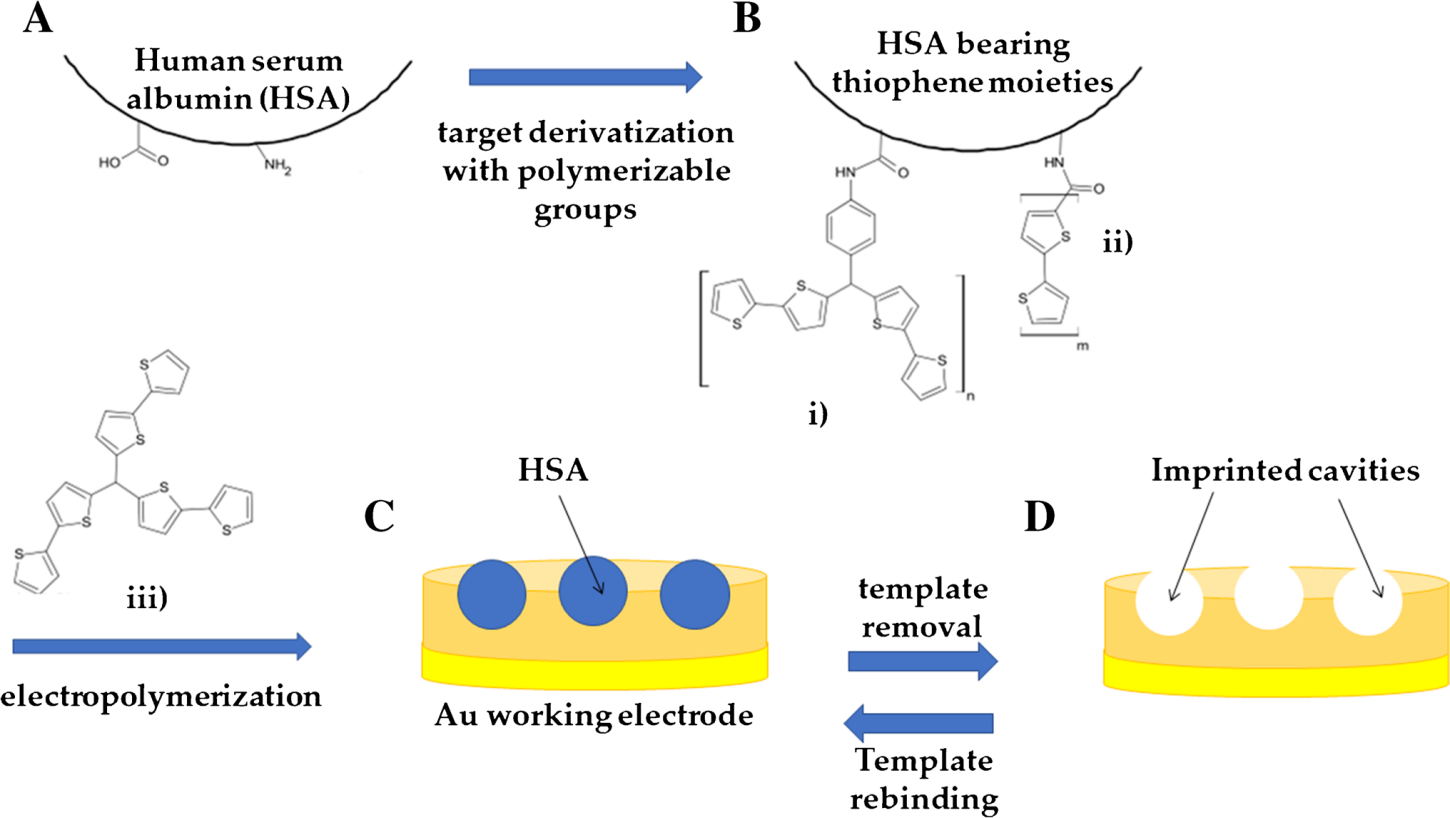
Synthesis scheme of a molecularly imprinted polymer film for HSA. (**A**) Target protein bearing –NH2 and –COOH groups was derivatized (**B**) with polymerizable bithiophene moieties (i) 2,2′- bithiophene-5-carboxylic acid and (ii) p-bis(2,2′-bithien-5-yl)methylalanine. (**C**) A MIP layer was directly electropolymerized on the electrode surface, using a solution containing labeled target and a cross-linker (iii) 5,5′,5″-methanetriyltris(2,2′-bithiophene). (**D**) The washing procedure produced the imprinted cavities. Adapted from [131]

Along with commonly used electropolymerizable monomers, other ad hoc derivatized agents have been exploited in order to introduce the desired functional groups, thus improving the interaction between the MIP and the chosen target. In this context, an excellent research activity was performed by Prof. Kutner’s group which developed several electro-synthesized MIPs by employing thiophene-derivative monomers with the aim of introducing ad hoc selected moieties within polythiophene backbone, enabling specific interactions with the target molecule [130–132]. In some cases, the authors derivatized the target, HSA protein, with bithiophene functional monomers for the development of an electrochemical [131] and an extended-gate field-effect transistor (EG-FET) [132] for the detection of HSA. They took advantage of the amino and carboxylic groups naturally present on the surface of the protein to graft on polymerizable functional groups, which enabled the synthesis of a highly selective polymer for the chosen target. The MIP-HSA film was directly prepared on the electrode surface by electropolymerization of a solution containing the derivatized target and other monomers (Fig. 13). In this procedure, the authors used a semi-covalent approach to protein imprinting that involves covalent binding of recognition moieties of functional monomers with functional groups of the template. After removal of the template (by immersing the MIP-HSA-coated electrode in NaOH solution for 45 min at 40 °C until the electrochemical response of the sensor was constant), producing the cleavage of the covalent bonds, unlike covalent imprinting, semi-covalent imprinting uses only non-covalent interactions (hydrogen bonds and/or via electrostatic, van der Waals, and hydrophobic interactions) in the rebinding step. The final sensor allowed the impedimetric detection of the target in the range of 4–80 μg mL^−1^. An extensive selectivity study was also reported. The sensor was found not to respond to most tested low-(molecular-weight) interferences including creatinine, urea, and uric acid. Nonetheless, the authors concluded that the presence of glucose should have no effect on the detection signal, as a blood sample must be diluted 1000-fold before HSA determination. Moreover, the sensor was practically insensitive to proteins such as CytC and Myo, while showing a certain response to Lyz. According to the authors, this was because nearly half of the Lyz sequence consists of random coils that are flexible. These could be responsible for Lyz fitting, at least partially, to the shape and locations of recognition sites within the imprinted cavities.

Besides the well-recognized advantages from MIP electropolymerization, some key aspects must be critically evaluated for achieving satisfactory imprinting performance.

Firstly, the possible redox activity of the target must be taken into strict consideration, as it can influence the MIP film electrodeposition process. The products of template redox reactions can interact with the forming polymer and/or be adsorbed on the electrode surface, leading to fouling phenomena and possibly reducing film adhesion to the electrode as well as further template electrochemical detection. Although such issues are quite limited in the case of biomacromolecule imprinting due to their limited electroactivity, a number of ploys have been proposed to overcome these problems, including the use of non-electroactive template analogues [133] and the production of resistive MIP self-barriers that prevent the redox processes of templates at the electrode [133, 134]. In a fairly recent work, Gonzalez-Vogel et al. attempted to develop a MIP for the detection of an electroactive lignin marker [135]. After checking the electroactivity of the template, a protective barrier of poly(3,4-ethylenedioxythiophene) (PEDOT) was first electropolymerized on the glassy carbon electrode surface before the MIP synthesis in order to prevent oxidation of the template molecule at the sensor surface. This made it possible to limit the redox activity of the target on the electrode surface (although not completely, as confirmed by the authors). The final sensor was able to detect the target in a range between 10^−6^ and 10^−2^ M. A better approach is to use electro-reducible functional and cross-linking monomers for imprinting of electro-oxidizable templates and vice versa. In this respect, a very interesting work came from Sharma et al. [136], who proposed the use of fullerene derivatives as reductively electroactive functional monomers for imprinting of adenosine-50-triphosphate (ATP), an electro-oxidizable template. The reductive electropolymerization was performed by potentiodynamic conditions, applying 12 potential cycles between 0 and −1.30 V versus Ag/AgCl at a sweep rate of 0.05 V/s. It was demonstrated that the application of such conditions did not produce any chemical or conformational changes in the target, and that the generation of reaction by-products was avoided. The sensor developed was able to determine this target up to a detection limit of 0.03 mM. Selectivity with respect to structural analogues of ATP was quite pronounced. That is, sensitivity to ATP was nearly nine times that to adenosine monophosphate, four times that to thymidine triphosphate and guanosine triphosphate, and nearly 2.5 times that to adenosine diphosphate. However, it was merely twice that to cytidine triphosphate (CTP). The latter was explained in view of the structural similarity of CTP and ATP with all the H-bond-forming groups of ATP also present in CTP. Moreover, sensitivity to adenine was approximately 15 times lower, while phosphate and guanosine did not interfere at all.

Secondly, another key aspect to consider in the electropolymerization of MIPs for macromolecules is the nature of the electrode material. It is generally accepted that proteins are variously adsorbed on surfaces with which they come into contact [137, 138] upon the instauration of van der Waals, hydrophobic, and electrostatic interactions [139], particularly on gold [127] and carbon-based [137] surfaces. Such a phenomenon was studied in a work by Zhang et al. [127] in the design of an electrochemical MIP-based sensor for the detection of transferrin (Tfr), a globular protein. The influence of the nonspecific binding of Tfr to the gold electrode surface was investigated by CV and SWV measurements in ferro/ferri-cyanide solution, with the aim of discriminating the contribution of the protein binding to the MIP from that related to adsorption on the surface of the electrode. Thus, changes in the redox probe signal on bare electrodes after exposure to target solutions at different concentrations were monitored to verify that Tfr was strongly adsorbed on the electrode surface as shown by suppression of the redox marker signal. It was hypothesized that this nonspecific adsorption may be due to the cysteine-rich domains of the globular proteins. The authors exploited this effect to obtain the adsorption of the protein before the electrosynthesis of a MIP layer using scopoletin as functional monomer. They then compared the performance of the resulting MIP with that of the MIP electro-synthesized from a monomer-template mixture. For both MIPs, they claimed that the variation in the analytical signal after rebinding was not only due to interactions between template and polymer, but that there was an important contribution related to the interactions between the protein and the gold surface, which was higher for proteins rich in cysteine residues. In addition, it was observed that the MIP synthesized around the adsorbed target was less selective than the other, possibly due to the unfolding of the target proteins upon the adsorption on the Au surface, which impairs the molecular imprinting process.

Nonspecific adsorption phenomena have also been confirmed, in particular for globular proteins due to their large size and multitude of functionalities [127, 137], on carbonaceous materials [137, 138, 140], resulting in a deterioration of sensor performance in the case of target protein detection or when the matrices studied contain proteins [140]. The strategy of preliminary anchoring of the target protein to the electrode surface prior to the electropolymerization [16, 37] in surface imprinting schemes (section 1.1) can reduce nonspecific adsorption phenomena. As discussed above, this approach also has the advantage of creating more uniform binding sites in the MIPs [37].

### MIP nanoparticles

The fruitful integration of the fields of imprinting and nanotechnology has certainly reached an apex with the synthesis of the imprinted material itself at nanoscale, of which MIP nanoparticles (MIP NPs) represent the mostly successful example.

Several strategies have been explored during recent years for the synthesis of MIP NPs, including precipitation [141–143], emulsion polymerization [144–146], core–shell grafting [125, 126, 147–149], and solid-phase synthesis [75, 150, 151]. Unlike bulk materials, MIP NPs have a higher surface-to-volume ratio and greater total active surface area per unit weight of polymer. Imprinted cavities are thus more easily accessible to the template, which improves binding kinetics and facilitates the template removal process, improving their overall performance [5, 152–154]. While all these fascinating features have allowed their successful applications in several fields including imaging [155, 156], spectroscopy [157], and sample treatments [158], when dealing with their use in sensing applications, an additional extra step has to be performed/optimized related to MIP NP immobilization on the electrode surfaces. As easily argued, this step has a fundamental role in the sensor assembly, as it has to promote the firm and homogeneous anchoring of the MIP NPs to the transducer surface while leaving their binding sites available for target interaction.

To this aim, two approaches are commonly proposed: (i) MIP NP incorporation within the electrode materials, and (ii) MIP NP immobilization on the electrode surface through the use of polymeric membranes acting as a scaffold and support structure [7, 13] or by a suitable linker (e.g. SAM) grafted on the electrode [150, 159, 160].

The first strategy can be followed when graphite-based electrodes or carbon paste electrodes are used, as MIP NPs are mixed with the components assembled to prepare the electrode [161]. Interestingly, in some cases the preliminary functionalization of electrode material with MIP is performed before assembling the electrode, as reported in a work by Yoshimi’s group [162] for the development of an electrochemical sensor for heparin determination in saline buffer and bovine blood. A heparin-imprinted copolymer was first grafted directly onto graphite particles by radical polymerization using two acrylamide-based monomers. The grafted particles were thoroughly mixed with silicon oil and ground into a paste in a polytetrafluoroethylene mortar. The as-modified graphite paste was then packed into the tip of a capillary to fabricate a MIP-functionalized carbon paste electrode. This sensor was able to selectively detect the target in a concentration range between 0 and 8 units mL^−1^. This approach certainly offers easy sensor assembly but could fail in guarantying MIP NP homogeneous distribution and, in turn, reproducible sensor responses. The limited application of this approach for the integration of MIP NPs with the electrode is probably a reflection of these limitations, particularly stringent in the case of macromolecule imprinting.

The second approach is more versatile and more widely used, allowing for the proper selection of the method for anchoring MIP NPs to the transducer depending on their characteristics. A simple method consists in preparing mixtures containing MIP particles and material acting as an adhesion network to the electrode surface, such as agarose [93, 163], chitosan [164], sol-gel, or acrylic derivatives [165–167], further deposited by drop- or spin-coating on the electrode surface. An example is provided by Yang and coworkers [93], who produced an electrochemical sensor for BSA protein using MIP particles synthesized by cryogenic polymerization of acrylamide-derivate compounds. A mixture of agarose and MIP NPs was dropped on the surface of a glassy carbon electrode and dried to obtain a solid membrane. The electrochemical sensor showed a linear response for a target logarithmic concentration range (10^−16^– 10^−12^ M).

One common approach involves anchoring of previously prepared MIP NPs onto the electrode surface using molecular linkers [150, 160]. This technique is particularly suitable with gold surfaces which are easily modified with thiol-bearing molecules exposing specific functionalities (typically amino- or carboxy-terminated) which can readily react with MIP NP moieties, often upon EDC/NHS coupling reactions. Such a functionalization scheme was reported by Garcia-Cruz et al. [160], who developed a sensor platform based on electro-responsive MIP NPs, capable of detecting different kinds of targets (small and high-molecular-weight compounds). MIP NPs were produced by solid-phase synthesis and then covalently attached to SPGEs using thioalkane linkers. For that, SPGEs were firstly incubated in a cysteamine ethanolic solution and then EDC/NHS chemistry was used to anchor the MIP NPs. A similar approach was proposed by Canfarotta et al. [150], who described the solid-phase synthesis of MIP NPs and their integration into a label-free capacitive sensor to detect trypsin. To immobilize MIP NPs on a gold working electrode, electropolymerization of tyramine was firstly performed in order to introduce free primary amino groups on the surface of the electrode. Later, the modified electrode was incubated in a glutaraldehyde solution to afford a linker between the amino groups exposed on the electrode and on MIP NPs. The as-developed sensor was able to detect minute amounts of the trypsin protein up to a concentration of 1.0 × 10^−14^ M [150]. Good results were also obtained in terms of sensor selectivity, which was tested against chymotrypsin, Lyz, BSA, and CytC. Selectivity coefficients were calculated from the ratio between the sensor response to the analyte and to the competitor molecule, obtaining satisfactory values ranging from 8.3 to 54.1, which evidenced the high selectivity of the developed system.

Finally, an original approach was proposed by Zhao and coworkers [90]. The authors explored the preparation of MIP NPs using an ad hoc synthesized amphiphilic copolymer as “macromonomer,” with complementary moieties to target molecules (BSA, OVA). MIP NPs were obtained by precipitation polymerization using a solution containing the macromonomer and targets, inducing their co-assembly. To prepare the imprinted sensor, NP solution was dropped onto a cleaned gold electrode, which was irradiated with UV light for 30 min to attach the nanoparticles to the electrode surface by “UV-curing.” The targets were indirectly revealed by monitoring the redox processes of an electrochemical probe ([Fe(CN)_6_]^3−/4-^), with BSA being detected in a range between 10^−14^ and 10^−9^ mg mL^−1^.

**Fig. 14:**
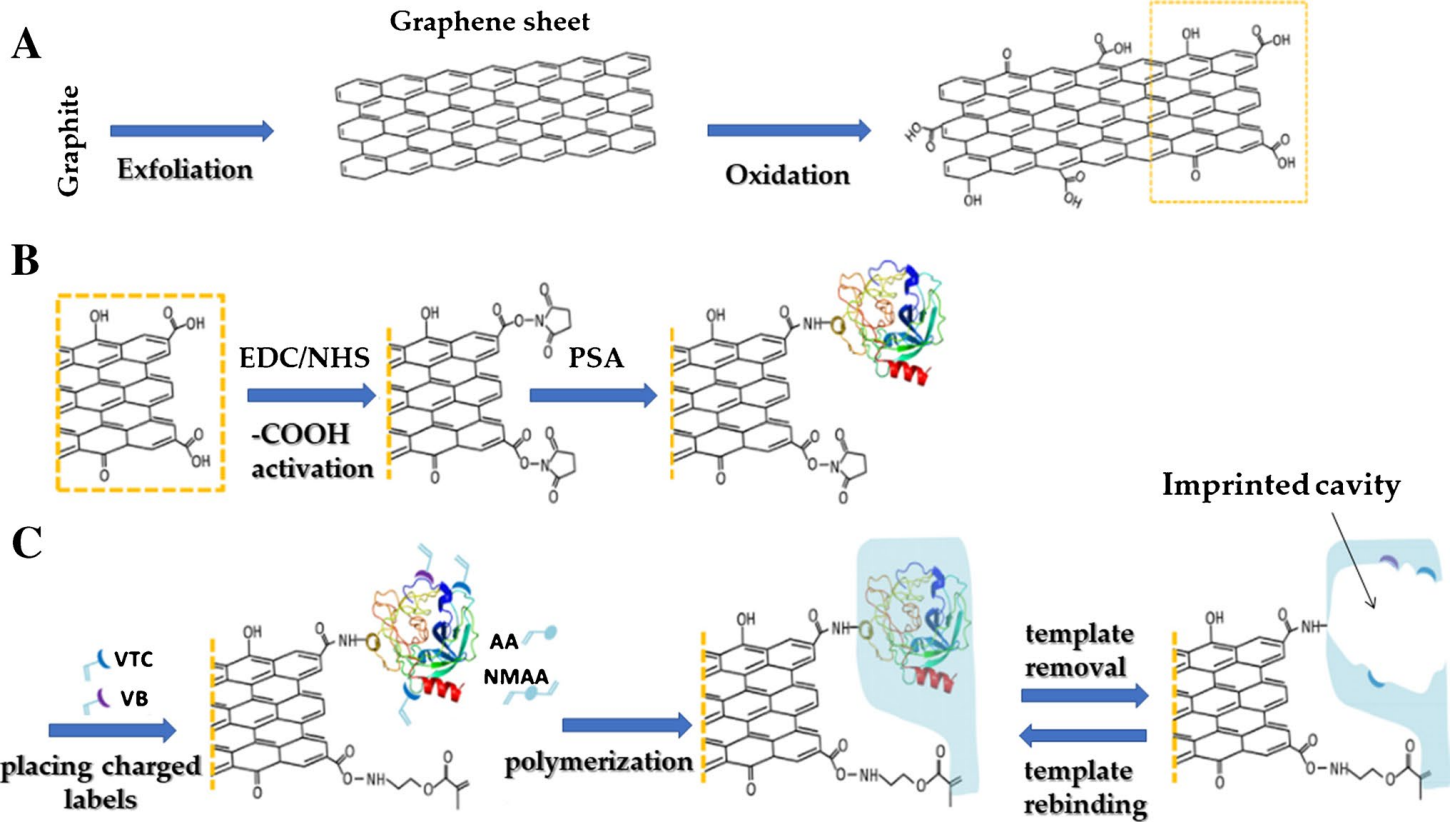
Scheme for synthesis of a MIP for PSA. (**A**) Sheets of graphene oxide (GO) were obtained by exfoliation and oxidation processes of graphite. (**B**) PSA was anchored on oxidated graphene sheets using EDC/NHS coupling reactions; (**C**) PSA anchored on GO sheets was labeled with polar monomers (VTC and BB). This obtained material was added to a solution containing other monomers (acrylamide, AA, and *N*,*N*-methylenebis(acrylamide), NMAA), whose polymerization was started by a radical initiator. Subsequent template removal produced imprinted cavities. Adapted from [172]

Along with MIP NPs, other examples of the fruitful coupling of nanotechnology and imprinting technology have been illustrated in several works exploiting the integration of MIP film with both metallic [92, 123, 168] and carbon-based [55, 88, 169, 170] nanomaterials in order to exploit their well-known properties in signal transduction, due to their increased surface area, remarkable conductivity, and excellent catalytic activity, all key elements in the design of an electrochemical sensor. The documented multifunctional properties of these nanomaterials, including high conductivity, interactive functions at the surface, and large surface-to-volume ratios, have indeed proved to increase sensing sensitivity [97, 98, 171–173]. An interesting work in this sense is that proposed by Moreira et al. [97], who developed a potentiometric sensor for troponin T (TnT) by synthesizing a MIP film on MWCNTs to produce nanostructured recognition elements. Later, these materials were dispersed in a poly(vinyl chloride) (PVC) matrix that acted as adhesion membrane on the surface of conductive wires used as working electrodes. The developed sensor detected the TnT at nanomolar levels, with a LOD equal to 160 ng mL^−1^. Another example in this context was provided by Rebelo et al. [172]. For MIP development, the authors copolymerized a mixture of monomers in the presence of the target (PSA), which was previously anchored to the surface of graphene sheets. In this case, the oxidation process of graphene was carried out to form surface-exposed functionalities (Fig. 14) responsible for PSA immobilization upon EDC/NHS coupling reactions. Charged commercially available monomers were selected, namely (vinylbenzyl)trimethylammonium (VTA) with a positive quaternary ammonium salt and vinyl benzoate (VB) with an ester function providing a negative polarity, which interacted with the negative and positive portions of the target molecule, respectively. The use of charged monomers resulted in a MIP with improved protein recognition ability compared to MIPs without charged monomers, due to stronger interactions with the charged portions of the target protein. The imprinted materials were mixed with the components of solid-contact carbon electrodes to prepare the final sensor, which was able to detect the protein up to concentrations of the order of nanograms per milliliter.

## Suitable electrochemical signals for MIP-mediated macromolecule recognition

**Fig. 15:**
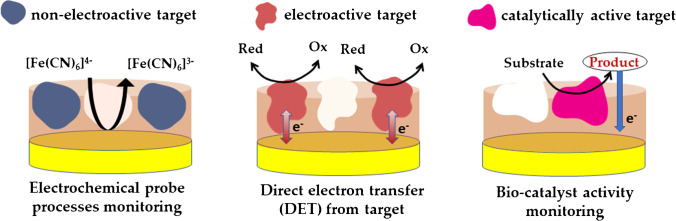
Different kinds of target protein and detection schemes in MIP-based electrochemical sensors

It is well known that the design of a good sensor implies that the target recognition event by the receptor (synthetic or not) is converted into an easily readable and usable signal [134, 174]. In the case of electrochemical sensors, a wide spectrum of transduction techniques are available, including voltammetry [86, 122, 131, 169], amperometry [175, 176], potentiometry [82, 172, 177, 178], impedimetry [169], and conductometry [178–180], which has certainly also contributed to increased research interest in their application in the integration with MIPs as recognition element. Although the choice of transduction is related to the nature of both polymer and target, in most cases it is the latter which mainly governs the selection of the transduction scheme. In particular, in the case of MIP-mediated macromolecule electrochemical detection, three major groups of targets can be distinguished: (i) non-electroactive proteins, (ii) template proteins carrying electroactive moieties, and (iii) catalytically active targets (e.g., enzymes or enzyme-labeled targets) [7, 17] (Fig. 15).

In general, when designing a MIP-based electrochemical sensor for protein, according to the intrinsic characteristics of the analyte, the transduction can thus be direct when the redox processes of the target itself and/or its redox-active products are monitored, or indirect when the target is not electroactive and the change in the signal of an external redox marker is monitored [7, 181]. Needless to say, in both cases, for quantitative analysis, it is necessary to establish a relationship between the concentration of the target and the change in the measured signal.

### Indirect transduction: the so-called gate effect

**Fig. 16:**
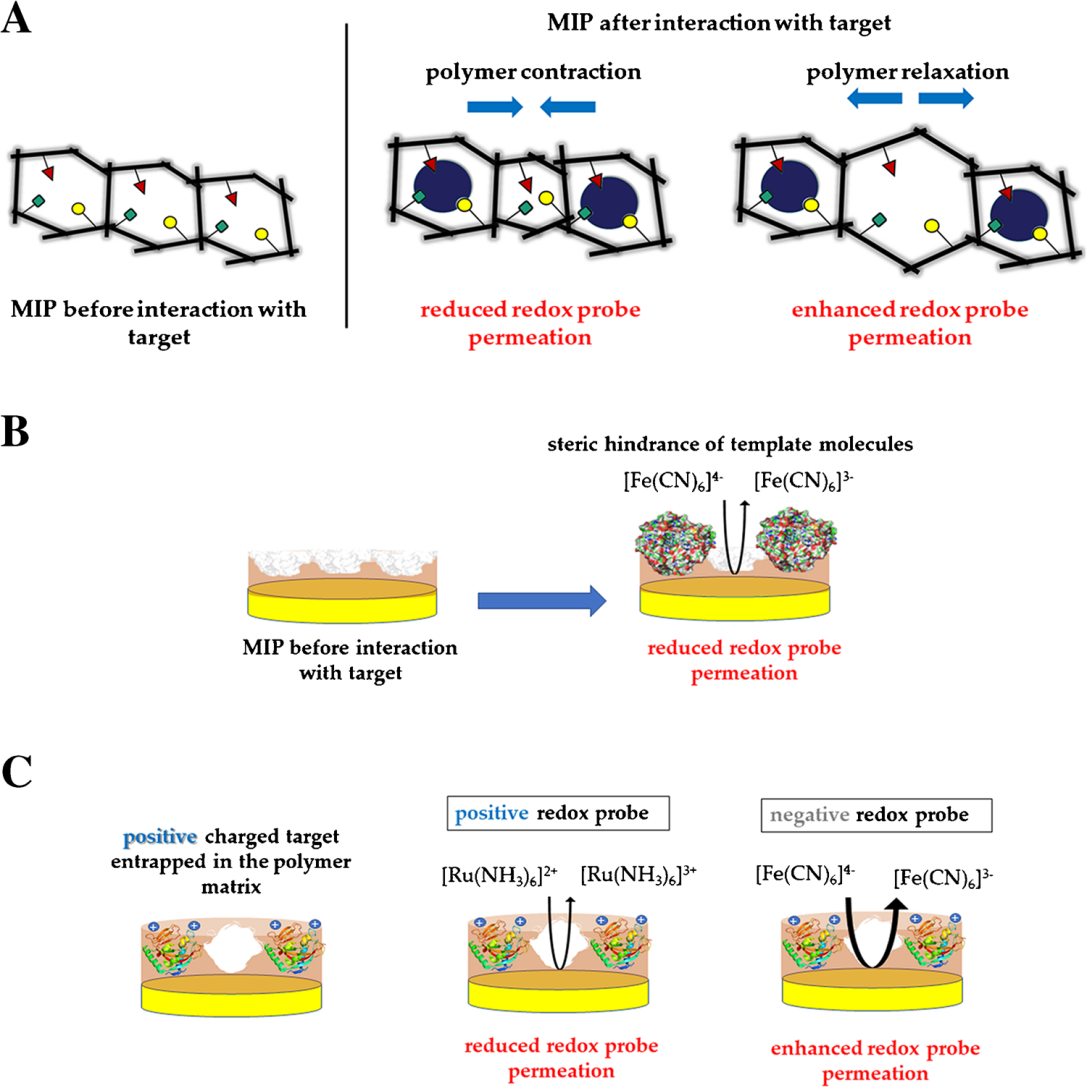
Gate effect mechanism. (**A**) Swelling or shrinking of the MIP film, (**B**) physical blocking of the redox probe diffusion, (**C**) charge-induced blocking of the redox probe diffusion

As mentioned above, when non-electroactive targets need to be detected, as frequently happens in the case of peptides and proteins, it is necessary to exploit redox processes of external redox probes. For this kind of transduction approach, the electrochemical readout is based on and is affected by the so-called gate effect [182]. It is known as a chemical-physical phenomenon occurring at the electrode–solution interface, related to changes in MIP features, especially its permeability toward the electrochemical probe, in response to the target recognition event by the MIP itself (Fig. 16).

After MIP synthesis, target removal from polymer matrix produces molecularly imprinted cavities through which redox marker permeation is possible because of its smaller size in comparison with the imprinted macromolecule. Subsequent interaction between polymer and analyte can produce both a change in the diffusion rate of the redox probe through the polymer film and a modification of the properties of the film itself, resulting in a variation of the signal related to the electrochemical marker [182].

Several works exploiting this mechanism have been published in the last decade [7, 89, 119, 153]. The indirect transduction approach is still widely used today, and sensors continue to be developed even for hot topic targets, such as those related to the current COVID-19 pandemic [21].

As schematically illustrated in Fig. 16A, a commonly observed phenomenon [182] consists in a swelling of the polymer as a consequence of the interaction between the MIP and target, with subsequent enlargement of the imprinted cavities, resulting in an increase in the permeability of the probe. Alternatively, and more frequently, the binding with the target leads to shrinking of the polymer with a decrease in faradic currents due to the hindered redox probe diffusion.

Although such approaches have been widely used in macromolecule MIP-based electrochemical detection, it is worth highlighting that particular attention should be paid when developing MIPs with polymeric materials prone to such shrinking/swelling phenomena, as modifications of detected current signals merely imputable to polymer permeability could be erroneously interpreted as due to the target binding process. This could occur particularly with polymers that are well known for their permeability properties, such as poly(phenylenediamine) (PPD) [183], which is widely used in the synthesis of MIPs exploiting such gate effect for electrochemical sensing [124, 184–186]. PPD films, especially those prepared by electropolymerization, have indeed been used in the past for the assembly of electrochemical sensors simply exploiting their permeability/permselectivity properties which were demonstrated to be significantly affected by polymerization conditions (e.g., potentiostatic or potentiodynamic deposition, pH). When using such polymers for the assembly of MIPs to be used in indirect electrochemical detection schemes, a preliminary careful study of polymer permeability should be done to evaluate whether it influences the sensor response regardless of the interaction with the analyte.

Another key aspect in the detection of macromolecular targets such as polypeptides and proteins is their steric hindrance. If large macromolecules occupy MIP binding sites, they may physically block the diffusion of redox marker molecules to the electrode surface (Fig. 16B) thus determining a decrease of recorded current [182, 187].

The effect of impeded redox marker diffusion through the imprinted matrix can arise not only from physical blocking but also from electrostatic repulsion due to the accumulated charge within the MIP matrix upon template rebinding. The MIP interaction with positively charged macromolecules can lead to an accumulation of positive charges, limiting the diffusion of redox species of the same charge (such as [Ru(NH_3_)_6_]^2+^/[Ru(NH_3_)_6_]^3+^). On the contrary, when the bound template is negatively charged, it can act to repel negatively charged electrochemical probes (eg., [Fe(CN)_6_]^3−^/[Fe(CN)_6_]^4−^) (Fig. 16C) [182]. On the basis of these considerations, electroactive probes can be properly selected in relation to the nature of the template and operating conditions (template protein isoelectric point, medium pH, etc.). Moreover, the interaction with charged macromolecule template can be promoted during MIP assembly by employing polymer backbone bearing charged functionalities, as discussed above (section 2) [172].

As mentioned above, the nature of the polymeric layer also influences the detection scheme to be adopted. Indeed, while the above-described mechanisms have been demonstrated to work well in the case of non-conductive MIP polymer, when dealing with conductive polymers, the polymer conductivity may represent an additional aspect to be taken into account, possibly playing an important role in the rebinding process. An interesting study by Kutner’s group [187] explained how the gate effect affects the electrochemical readout in the case of MIP conductive films, using a polythiophene-based MIP and *p*-synephrine (SYN) as target molecule. Under these conditions, it seems that a crucial factor is the modification of the electrochemical properties of the polymer film, with particular reference to its conductivity due to variation in the mobility of delocalized charges on the polymer backbone [187]. In particular, the authors demonstrated that the observed decrease in the peak current for both positively and negatively charged redox probes with the increase in the SYN concentration did not originate from swelling or shrinking of the MIP film. Instead, it was caused by the decrease in the electrochemical reversibility of the redox probe electrooxidation. This effect was attributed to the plausible decrease in radical cation (polaron) mobility in the conductive MIP film [119, 187, 188].

The classical approach for exploiting the gate effect in MIP-based electrochemical sensors for protein consists in exposing the MIP sensor to solutions with different concentrations of target and then evaluating possibly related signal variation in the electrochemical probe. There are several examples reported in the literature (Table 2), with voltammetry and electrochemical impedance spectroscopy (EIS) [37, 65, 85] being the most widely used signal transduction techniques. As far as the choice of transduction technique, it can be highlighted that voltammetric techniques are suitable when an appreciable peak current is observed after the exposure of the MIP to the probe molecule, whose modification upon rebinding is then used as analytical signal. EIS detection, on the other hand, can also be used when such a situation does not occur—i.e., a low initial current peak is recorded, possibly due to limited redox probe permeation within the imprinted cavities. This is because the analytical signal commonly used in EIS is the charge-transfer resistance associated with the process by which electrons are exchanged at the solution–electrode interface. It is thus affected by surface changes that enhance or hinder the electron transfer and can be used to monitor the ongoing redox processes, without being directly related to redox molecular diffusion through the MIP layer.

**Table 2:** Transduction approaches and electrochemical readout for some representative electrochemical MIP sensors

Transduction approach	Analyte	Electrochemical readout	Redox processes/signal by	Ref.
*Indirect* determination	Lyz	EIS	[Fe(CN_6_)]^3−/4−^	[37]
	CA-125	DPV	[Fe(CN_6_)]^3−/4−^	[85]
	HSA	DPV EIS	[Fe(CN_6_)]^3−/4−^	[131]
	HTHP	CV	[Ru(NH_3_)_6_]^2+/3+^	[189]
	NSE	CV DPV	[Fe(CN_6_)]^3−/4−^	[65]
	Protein A	EIS	[Fe(CN_6_)]^3−/4−^	[169]
	PSA	DPV	[Fe(CN_6_)]^3−/4−^	[88]
	23H-Porphine tetratosylate (Por)	CV	[Ru(NH_3_)_6_]^2+/3+^	[190]
	Troponin T	DPV	[Fe(CN_6_)]^3−/4−^	[122]
*Direct* determination	CytC	CV	DET	[39]
	Acetylcholinesterase	AMP	Target products	[34]
	BSA	CV	DET	[96]
	HTHP	CV DPV	DET	[189]
	Tyrosinase	AMP	Target products	[87]
	Recombinant human erythropoietin (rhEPO)	Potentiometry	Target	[191]
	HSA	Potentiometry	Target	[132]

Abbreviations: CV: cyclic voltammetry; EIS: electrochemical impedance spectroscopy; DPV: differential pulse voltammetry; SWV: square wave voltammetry; AMP: amperometry; DET: direct electron transfer

Ma et al. [88] prepared a voltammetric sensor for tumor marker (PSA) detection, functionalizing glassy carbon electrodes with graphene nanoplatelets, gold nanoparticles, and chitosan, before immobilization of the protein target and polymer synthesis. PSA was indirectly determined by DPV, achieving a very low LOD (0.15 pg mL^−1^). In another study, Pacheco et al. developed an electrochemical sensor equipped with a MIP film, which was electro-synthesized using a solution containing the monomer and the target. Also, in this case, indirect template detection was performed by DPV, monitoring ferri/ferrocyanide probe current changes in the MIP after exposure to solutions containing the target HER2-ECD, a breast cancer biomarker protein [192]. In another study [85], a sensor for the detection of a mucin-like glycoprotein, CA-125, was developed. Indirect transduction was used in this case as well. In particular, after the synthesis, the MIP was exposed to a solution containing both the target and the marker redox, and then the signal of the electrochemical probe was monitored by SWV to assess sensor modifications induced by the MIP–target interaction. It was observed that the presence of CA-125 in the redox probe solution increased the anodic peak current at ~0.2 V, proportionally to its concentration, and was thus used as the analytical parameter to quantify the CA-125: a linear trend was observed from 0.01 to 500 U mL^−1^. However, it was not exhaustively explained by the authors how the target affected the electrochemical reading.

As mentioned above, the reliability of the results when using such an approach may suffer from possible variations in the experimental conditions (ionic strength and/or pH, solvents) during MIP assembly/testing, possibly leading to misleading current signal modifications [119], being ascribed to factors not directly related to analyte exposure but instead to polymer flexibility and to swelling/shrinking processes [193]. Some authors have indeed exploited the polymer swelling effect under certain conditions in order to incorporate a greater amount of template into the polymer matrix, possibly resulting in a higher number of binding sites in the final MIP [194].

As an alternative to the common use of the redox marker, some research groups have successfully incorporated the direct probe into the imprinted polymer matrix [195]. Subsequently, this approach was successfully applied to MIP NPs prepared by solid-phase synthesis. Mazzotta et al. [195] developed an electrochemical sensor for the recognition of vancomycin using MIP NPs produced testing two different ferrocene-derivative monomers (namely, vinylferrocene and ferrocenylmethyl methacrylate) added in different amounts to a polymerization mixture. Under optimized conditions, the indirect electrochemical detection of vancomycin was enabled by the change in the ferrocene group redox properties upon the exposure to vancomycin. According to the authors, the observed behavior was attributable to the impedance of the electron transfer process of the ferrocene redox sites within nanoparticles by their interaction with non-electroactive vancomycin. The sensor was able to selectively detect the target analyte in a linear range between 83 and 410 μM. After this first successful application, other works have reported the use of nanoMIP integrating both recognition and reporting functions by synthesizing MIP NPs tagged with a redox probe [196]. Although this alternative indirect electrochemical detection scheme proved to be effective for different imprinted targets, to the best of our knowledge, it has not yet been explored for protein detection. Such a strategy could represent a useful approach for the imprinting of macromolecules when the electrochemical signal of an external probe cannot be used, as for example in tests in vivo. In such cases, the two steps (namely, incubation with the target protein and subsequent signal recording in the probe solution) cannot be performed separately. The possibility of having an imprinted polymer electroactive per se could be highly beneficial for expanding MIP applications. Moreover, in the case that the inclusion of electroactive moieties within the polymer is not feasible in the adopted synthesis conditions, post-synthetic derivatization approaches for introducing the desired electroactive functionalities in the recognition cavities could be developed.

### Direct electrochemical transduction: the case of redox proteins and catalytically active targets

When designing MIP sensors for electroactive targets, the electrochemical signal is commonly related to the direct electron transfer (DET) from the analyte to the electrode originating from redox processes involving the target (Fig. 15). In this case, therefore, there is a “direct conversion” of the analyte into an electrochemically usable signal [119, 197].

While this approach has been widely exploited for MIP-mediated sensing of small molecules [121, 163, 198, 199], its application to the detection of macromolecules such as peptides and proteins appears restricted to a small number of examples [197]. This is due to their bulky size which makes their redox centers (if they have any) not easily accessible, thus making them less likely to be eligible for DET [200–202]. To promote DET, the proteins must be in an “electroactive orientation” with respect to the electrode, which means that their redox active moieties should be oriented toward the surface of the electrode in order to facilitate the electron transfer [201]. When the proteins adsorb in an orientation that does not allow the “direct contact” between the active sites and the electrode, or if the proteins do not adsorb on the surface at all, the direct electron transfer is not possible, because the excessive distance does not allow it [200].

Direct transduction can be performed instead in the case of metalloproteins and proteins bearing redox centers, through which the direct exchange of electrons with the electrode is possible. When in the polymer matrix, if the protein is in a “productive orientation” [39], with the redox centers toward the electrode, upon electrical stimulation through the application of an electrical potential, a charge transfer from the target to the electrode surface can be observed [17], resulting in a signal suitable for the quantitative evaluation of the protein. However, examples are very limited [39, 189]. Bosserdt et al. [39] developed an electrochemical sensor for the detection of a small-membrane heme protein, cytC, electrodepositing a non-conductive polymer film from an aqueous solution of scopoletin and cytC on the surface of a gold electrode previously modified with a SAM of mercaptoundecanoic acid (MUA). The “active site” responsible for DET in cytC is an iron porphyrin (heme) covalently linked to the rest of the protein by thioether bonds. The modification of the iron redox state between Fe^2+^/Fe^3+^ due to the electron transfer can be monitored to confirm the occurrence of the MIP–cytC interaction. Specifically, in this work, cyclic voltammetry was performed for DET measurement between cytC and the modified electrodes to obtain information about both the conformational state of cytC (monitoring the change in the formal potential) and the amount of rebounded cytC (by evaluation of the surface coverage). The sensor was able to quantify the target, showing good selectivity for BSA, Myo, and Lyz. The same group in another work [189] described an interesting MIP sensor for hexameric tyrosine-coordinated heme protein (HTHP), a heme protein, which exhibits an intrinsic peroxidase-like activity. It was observed that the MIP was able to determine the analyte by DET. HTHP has a hexameric ring structure, and each of the six monomers contains one non-covalently bound heme in a hydrophobic pocket and the iron is coordinated by tyrosine in the proximal side. Also, in this case, for DET evaluation the redox processes of the Fe^2+^/Fe^3+^ couple were used. Moreover, it was demonstrated that the enzyme entrapped in the polymer matrix retained its ability for electrocatalysis of hydrogen peroxide. The sensor showed good selectivity and preferentially bound the target over cytC, with a recorded imprinting factor of 12.

Another example of DET in a MIP-based sensor for protein was proposed by Reddy et al. [96]. In this work, glassy carbon electrodes were functionalized by drop-casting a hydrogel-based MIP which could selectively recognize the Hb. Although it is reported [202, 203] that hemoglobin can undergo DET under certain conditions, the “real” target was oxyhemoglobin, the Hb form bound to the molecular oxygen. The authors reported that this protein undergoes conformational changes, similar to those induced by pH change, due to its interaction with the imprinted cavities, which allows an electrochemical signal to be generated as a consequence of the direct electrochemical reduction of bonded oxygen in oxyhemoglobin during cyclic voltammetry.

For the development of MIP-based electrochemical sensors for enzymes and/or catalytically active macromolecules, the detection can be performed by directly measuring the enzymatic activity of biocatalysts bound to the polymer matrix. In practice, the electrochemical detection of redox products resulting from enzymatic processes enables target quantification. This type of approach has been successfully used for a variety of enzymes [34, 87, 204]. For instance, Jetzschmann et al. [34] developed MIP nanofilms for acetylcholinesterase (AchE) recognition monitoring of the rebinding of the template via the generation of thiocholine from acetylthiocholine, which was oxidized at the underlying gold electrode. Similarly, Yarman [87] developed a MIP sensor able to recognize tyrosinase, exploiting its catalytic activity. An amperometric detection was performed as follows: At −100 mV, o-quinone, which was formed by the enzymatic reaction, was reduced after stepwise addition of catechol in the working solution. The current produced by this process was used as analytical parameter for target detection. The MIP sensor had a linear range up to 50 nM, with a LOD of 4 nM. Moreover, the signal suppression by tyrosinase was 3.5-fold and 2.5-fold higher than that for BSA and CytC, respectively. The response observed to such interference proteins was ascribed to their smaller size than the target. In addition, it was found that ferritin, which is larger than tyrosinase, did not bind to MIPs, although no experimental data were shown.

Regardless of the redox nature of the target, protein electrochemical detection by MIP-based sensors can also be achieved by potentiometric measurements. When charged proteins bind to the surface of a thin MIP layer, there is a change in the surface potential, which can be conveniently used as an analytical parameter for target detection [132, 181, 191, 205, 206]. Considering that proteins in aqueous solutions have a net electrical charge whose magnitude depends on the isoelectric point of the protein and the ionic composition of the solution, it is possible to improve the performance of such potentiometric sensors by acting on these parameters [181]. Moreover, in potentiometry it is not necessary for the template molecules to permeate the MIP membrane up to the electrode surface, and thus there is no size constraint on the targets [206].

Some sensors for proteins have been successful developed by exploiting this sensing mechanism, but it should be noted that the imprinting process can be tricky, since proteins easily undergo conformational changes resulting in a modification of their multiple charge locations [206]. This issue has been addressed in various reports [172, 205]. Yu et al. [82] proposed a MIP film for CEA, obtained by deposition of a solution containing the target and a thiol (11-mercapto-1-undecanol). It seems that this simple procedure enabled the formation of a polymeric layer able to recognize the CEA by potentiometry. The optimized sensor showed a detection limit of 0.5 ng mL^−1^ and good selectivity, tested against matrix metalloproteinase-7 (MMP-7) and BSA. MMP-7 was selected as tumor marker with similar size as CEA, while BSA was tested, as in several other works, as the most abundant blood plasma protein in mammals. No response was observed in the absence of CEA, but a quite limited spectrum of potential interference molecules was tested.

For exploiting protein recognition based on the charge effect, the integration of MIPs with FET systems is also possible. A recent example is that proposed by Dabrowski et al., who functionalized an EG-FET with MIP film for sensitive determination of HSA [132]. The EG-FET transduction is sensitive to changes in the charge concentration near the FET extended gate, which influences the current passing between the source and drain of the transistor. Therefore, binding of charged species, such as proteins, to the MIP film deposited on the surface of the transistor’s extended gate leads to a change in the source–drain current proportional to the accumulated charge. The authors exploited the highly charged state of the target protein in solutions of pH far from its isoelectric points in order to achieve highly sensitive HSA determination by such transduction method, which was able to reveal the target at femtomolar levels.

## Conclusions

Research activity focused on the imprinting of macromolecules, such as peptides, proteins, and enzymes, has experienced very rapid growth over the past several years. This is due to the increased popularity of molecularly imprinted polymers, which during the recent decades have confirmed their ability to act as artificial antibodies, and also to the increased need for macromolecules as markers for monitoring samples and processes, as widely happens nowadays in the clinical, environmental, and biotechnological fields, with low-cost, robust, and simple-to-use devices. Both phenomena can be ascribed to the ever-wider application of MIPs as chemical sensors for macromolecules, which represent over 70% of the research activity devoted to the field of macromolecule imprinting (Scopus, December 2021). In particular, electrochemical transduction has been reviewed herein, illustrating the approaches used for MIP integration with the transducer surface, the physical formats suitable for MIP synthesis, and the strategies for generating readable analytical signals upon MIP–macromolecule interaction. Also, drawbacks inherent in macromolecule imprinting are highlighted, as well as key aspects to be taken into account when developing electrochemical imprinting of macromolecules for achieving a significant and reliable imprinting effect.

Despite the remarkable success of macromolecular imprinting technology in recent decades, several challenges and opportunities are still in front of researchers working in this field:

- The understanding of interactions responsible for recognition properties, of paramount importance for successful imprinting, could be improved by a more extensive use of bioinformatics, especially in the case of epitope imprinting.
- The use of organic solvents in the preparation of MIPs, typically employed in aqueous matrices, can represent a drawback. Although only rarely proposed in the case of electrochemical sensors, it can be at times required by the reduced solubility of functional monomer(s) in aqueous solutions. The exploitation of ionic liquids should be regarded as an interesting alternative.
- Increasing MIP specificity by inclusion of other element like aptamers in the recognition sites should be pursued. Such a hybrid approach could enhance MIP selectivity, which is in some cases limited, especially against smaller molecules and/or proteins with high structural similarity, possibly coexisting with the analyte in the real matrices.
- The application of post-imprinting functionalization (by electroactive groups) of the recognition cavities can represent an alternative way of generating a readable electrochemical signal and can further promote the integration of MIPs in existing biological analysis platforms in place of natural counterparts.
- Surface imprinting and nanoparticle formats should be further applied for improving the kinetics of mass transfer.
- The issue of reproducibility in preparation scale-up represents a barrier to commercialization. In this respect, the application of chemometric tools of experimental design for preparation optimization can be of great help.

The last of these represents a key point for extending the application of the imprinting technology from the research field to food, environmental, and pharmaceutical industries and to clinical diagnosis. This further improvement is already promoted by companies commercializing MIPs for different applications including sensors, such as MIP Diagnostics (UK) (https://www.mip-dx.com), Semorex Technologies Ltd. (Israel) (www.semorex.com), and NanoMyp (Spain) (www.nanomyp.com). Nevertheless, the commercialization of MIPs and MIP-based sensing devices is still in its early stage, especially if compared with the market of sensing platforms based on natural receptors. Research and industry should proceed in a synergistic way for realizing a technology that must be robust, reliable, cost-competitive, and easily scalable. This will enable significant growth in the field macromolecular imprinting and will open up a new path for its success in practical real-world applications and in everyday life.

## Abbreviations list

AAm: acrylic amide
AchE: acetylcholinesterase
ATP: adenosine-50-triphosphate
BDNF: brain-derived neurotrophic factor
BHb: bovine hemoglobin
BSA: bovine serum albumin
CA-125: carbohydrate antigen 125
CEA: carcinoembryonic antigen
CLRP: controlled/living radical polymerization
CS: chitosan
cTnT: cyclic troponin T
CV: cyclic voltammetry
CytC: cytochrome C
DEAEM: diethyl-aminoethyl methacrylate
DET: direct electron transfer
DPV: differential pulse voltammetry
eATRP: electrochemically mediated atom transfer radical polymerization
EDC: 1-ethyl-3-(3-dimethylaminopropyl)carbodiimide
EG-FET: extended-gate field-effect transistor
EIS: electrochemical impedance spectroscopy
Fer: ferritin
Hb: hemoglobin
HHb: human hemoglobin
HSA: human serum albumin
HTHP: hexameric tyrosine-coordinated heme protein
IgG: immunoglobulin G
LOD: limit of detection
Lyz: lysozyme
MAA: methacrylic acid
MAT: multi-step amperometry technique
MBA: *N*,*N*′-methylene-dipropylene amide
MBAAm: methylene-*N*,*N*-bis(acrylamide)
MIP: molecularly imprinted polymer
MIRATE: MIps RATional dEsign science gateway
MNPs: magnetic nanoparticles
MUA: mercaptoundecanoic acid
MWCNTs: multi-walled carbon nanotubes
Myo: myoglobin
NHS: *N*-hydroxysuccinimide
NIP: non-imprinted polymer
NIPAAm: *N*-isopropylacrylamide
NPs: nanoparticles
NSE: neuron-specific enolase
o-PD: o-phenylenediamine
OVA: ovalbumin
PEDOT: poly(3,4-ethylenedioxythiophene)
PGE: pencil graphite electrode
PSA: prostate-specific antigen
PVC: poly(vinyl chloride)
QCM: quartz crystal microbalance
SAM: self-assembled monolayer
SPCE: screen-printed carbon electrode
SPGE: screen-printed gold electrode
SWV: square wave voltammetry
SYN: *p*-synephrine
TnT: troponin T
Trf: transferrin
VB: vinyl benzoate
VTA: (vinylbenzyl)trimethylammonium
